# High‐Performance Deep Learning for Instant Pest and Disease Detection in Precision Agriculture

**DOI:** 10.1002/fsn3.70963

**Published:** 2025-09-15

**Authors:** Muhammad Bilal, Asghar Ali Shah, Sagheer Abbas, Muhammad Adnan Khan

**Affiliations:** ^1^ Riphah School of Computing & Innovation, Faculty of Computing Riphah International University Lahore Pakistan; ^2^ Department of Computer Science Kateb University Kabul Afghanistan; ^3^ Prince Mohammad Bin Fahad University Dhahran Saudi Arabia; ^4^ Department of Software, Faculty of Artificial Intelligence and Software Gachon University Seongnam‐si Republic of Korea

**Keywords:** crop disease, deep learning, fusion model, image processing, MobileNet and EfficientNet, pest detection, transfer learning

## Abstract

Global farm productivity is constantly under attack from pests and diseases, resulting in massive crop loss and food insecurity. Manual scouting, expert estimation, and laboratory‐based microscopy are time‐consuming, prone to human error, and labor‐intensive. Although traditional machine learning classifiers such as SVM, Random Forest, and Decision Trees provide better accuracy, they are not field deployable. This article presents a high‐performance deep learning fusion model using MobileNetV2 and EfficientNetB0 for real‐time detection of pests and diseases in precision farming. The model, trained on the CCMT dataset (24,881 original and 102,976 augmented images in 22 classes of cashew, cassava, maize, and tomato crops), attained a global accuracy of 89.5%, precision and recall of 95.68%, F1‐score of 95.67%, and ROC‐AUC of 0.95. For supporting deployment in edge environments, methods such as quantization, pruning, and knowledge distillation were employed to decrease inference time to below 10 ms per image. The suggested model is superior to baseline CNN models, including ResNet‐50 (81.25%), VGG‐16 (83.10%), and other edge lightweight models (83.00%). The optimized model is run on low‐power devices such as smartphones, Raspberry Pi, and farm drones without the need for cloud computing, allowing real‐time detection in far‐off fields. Field trials using drones validated rapid image capture and inference performance. This study delivers a scalable, cost‐effective, and accurate early pest and disease detection framework for sustainable agriculture and supporting food security at the global level. The model has been successfully implemented with TensorFlow Lite within Android applications and Raspberry Pi systems.

## Graphical abstract

This paper introduces a high‐performance Deep Learning fusion model that combines MobileNetV2 and EfficientNetB0 to conduct real‐time crop pest and disease detection. When trained on the CCMT dataset, the model attained 95.68% precision, 95.67% F1‐score, and a global accuracy of 89.12%. Designed to run smarter inference on low‐power edge devices such as smartphones and drones, it is cost‐efficient and scalable with a sustainable solution to agriculture.

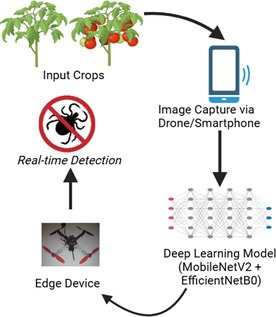

## 1 Introduction

The world is now facing pressure to ensure that there is enough food supply to sustain its growing population, and this is due to changing climatic forces. As a result, it is expected that the world population will be 9.7 billion by the year 2050, a fact that puts a heavy burden on agricultural productivity and sustainability (Gupta et al. 2023). One of the disruptive strategies introduced in the agriculture business is a precision agriculture method that applies digital systems to improve farming processes and resource optimization (Alam 2023).

The high prevalence of crop pests and diseases is one of the greatest concerns to crop yield and crop quality as the crop losses that occur due to this factor amount to around 40% of the total crops lost annually around the globe (Chen et al. 2022). Conventional sensing methods, for example, manual scouting and lab analysis are slow, labor‐intensive, and subjective and liable to error (Wang et al. 2023; Zhang et al. 2023).

This poses a serious concern, which is that existing methods of detection of crop diseases are not as effective as real‐time implementation, particularly in a low‐resource setting. An efficient, portable, and quality system is the need of the hour to fill this void. More so, the late detection is responded to by excessive use of pesticides that lead to population of the environment and health hazard.

Over the past years, deep learning (DL) models, specifically Convolutional Neural Networks (CNNs) have achieved immense success in automating the classification and detection of diseases and pests based on observation of an image (Xie et al. 2022; Hao, 2019; Zhiying 2019). Nevertheless, a great number of them need intensive computing resources, which do not make it possible to implement in the field (real‐time conditions: particularly rural sites with limited infrastructure). These barriers are doubly overcome by lightweight models such as MobileNetV2 and EfficientNetB0 because they offer precise detection results at a lower computational cost (Ahmed, Rashid, and Khan 2022). Different forms of deep learning use supervised learning methods because models require labeled data for their operation (Li et al. 2022).

**FIGURE 1:**
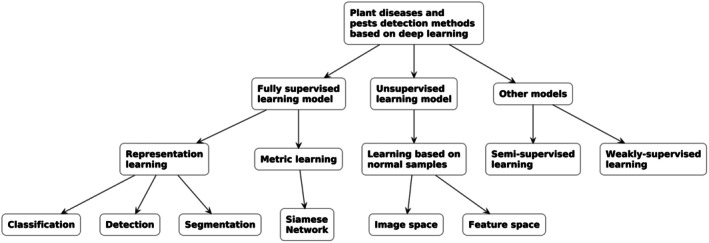
Taxonomy of deep learning‐based plant disease and pest detection methods.

The graphics in Figure 1 display machine learning types alongside supervised learning as the core component of DL.

There are three types of machine learning: supervised, unsupervised, and reinforcement. Figure 1 describes different machine learning and deep learning techniques applied for plant pest and disease detection and identifies three major approaches: fully supervised learning, unsupervised learning, and others, such as semi‐supervised and weakly supervised learning. Fully supervised models are trained with labeled data, where each image is labeled with a disease or pest. These encompass representation learning, extracting features to enhance classification, detection, and segmentation, and metric learning, for example, Siamese networks, which match image similarity to identify visually similar diseases. Unsupervised models require no labeled data and instead identify patterns by clustering similar images, learning from healthy samples to detect anomalies. Methods like feature space and image space learning assist in grouping and comparison. Other models, like semi‐supervised learning, use both labeled and unlabeled data to reduce labeling needs while maintaining performance. Weakly supervised learning employs coarse labels, including marking just whether a plant is healthy or diseased. This classification illustrates that deep learning techniques applied to pest and disease detection are supervised, unsupervised, or hybrid. Though supervised models excel with big, labeled datasets, unsupervised and semi‐supervised models are most apt for real‐world applications where labeling is restricted. The research takes a hybrid model from MobileNetV2 and EfficientNetB0, intending to deliver high precision while being computationally efficient

**FIGURE 2:**
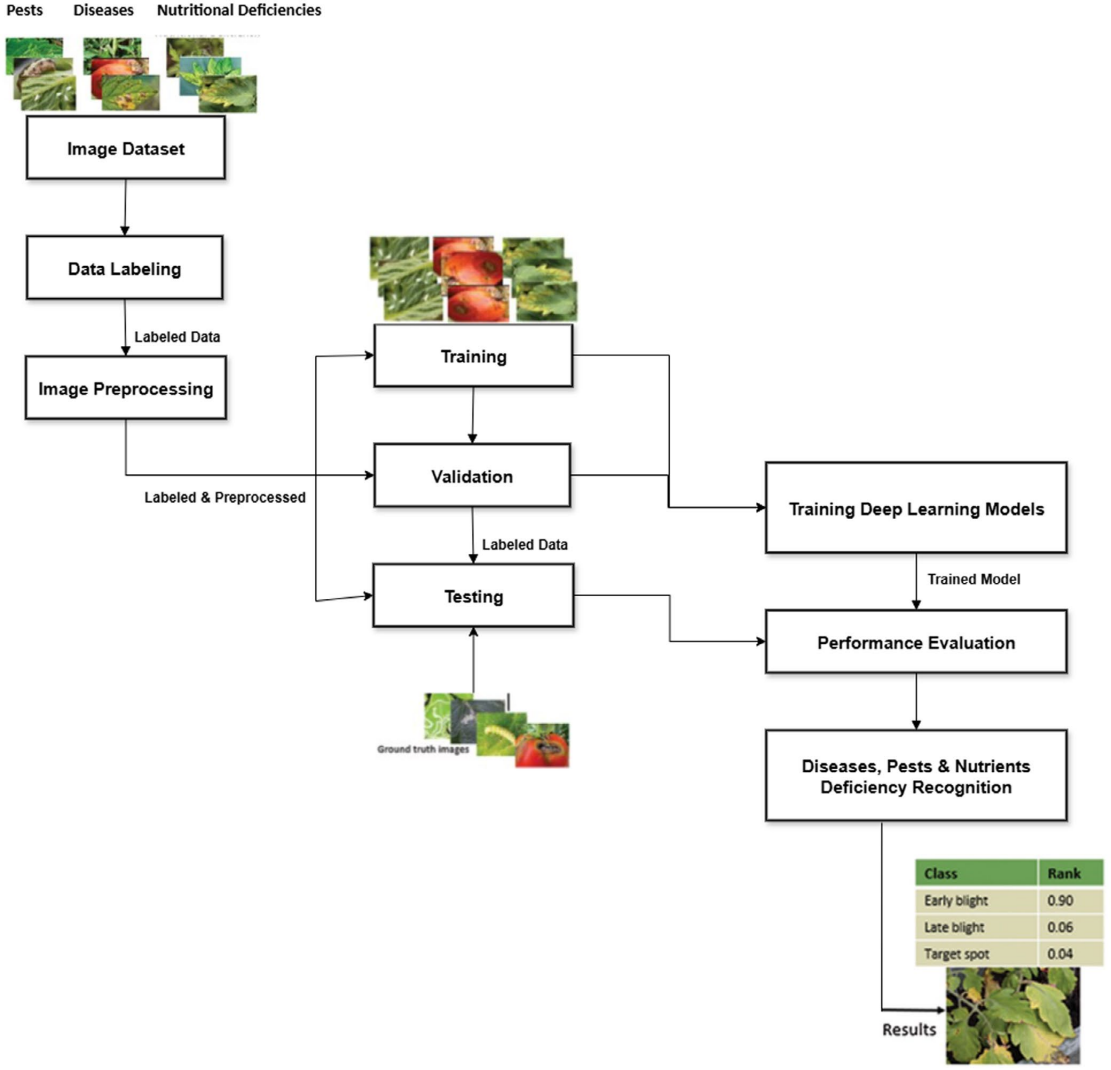
Workflow of deep learning‐based plant disease and pest detection.

Figure 2 describes an end‐to‐end deep learning pipeline for plant disease, pest, and nutrient deficiency detection. The process starts with Data Collection, where images are collected from research institutions, drones, and smartphones. Images are then labeled to represent the precise conditions correctly, forming a ground truth dataset. The Preprocessing phase improves image quality by resizing, noise reduction, normalization, and augmentation to enhance model robustness.

The data set is subsequently divided into Training, Validation, and Testing Sets, where training data is utilized for training the model, validation data for fine‐tuning it, and testing data for performance estimation on unknown inputs. While doing Model Training, architectures such as MobileNetV2 and EfficientNetB0 are used to train image features. The performance of the model is then estimated via Performance Evaluation by making use of measurements such as precision, recall, F1‐score, and confusion matrices for identification of misclassifications and the right generalization.

In the last Recognition Phase, the learned model predicts new plant images, giving labels and confidence values, allowing for prompt and well‐informed agricultural decision‐making. Their deployment on edge devices facilitates enhanced real‐time diagnosis, especially in remote regions where internet connectivity is poor, owing to Edge Computing and IoT technology developments (Jha et al. 2022).

This pipeline solves the crucial agricultural problem caused by pests and diseases, responsible for 40% of worldwide annual crop loss (Kumar et al. 2023). The traditional techniques tend to be less efficient and damaging to the environment (Singh et al. 2023). Precision Agriculture, which relies on remote sensing and CNN models such as YOLO and ResNet, provides real‐time monitoring capacity (Mishra et al. 2023; Liu et al. 2023). Nevertheless, due to high computational requirements, their deployment is constrained in rural areas. To overcome this constraint, the paper presents High‐Performance deep learning models employing knowledge distillation and quantization. The models remain highly accurate yet optimized for low‐resource conditions to facilitate scalability and access by smallholder farmers (Akbar et al. 2024).

This study contributes the following: To conclude, this paper proposes a new fusion model that uses MobileNetV2 and EfficientNetB0 combined with the quantization and pruning methods adjusted to avoid the latency of deployment to an edge device.

1. The progress in a new model of fusion between MobileNetV2 and EfficientNetB0 fused models with increased classification accuracy and latency reduction.
2. Application of edge‐compatible methods such as pruning of models, quantization, and knowledge distillation in real‐time deployment.
3. Reduction in the degree of class imbalance via SMOTE and weighted loss functions to enhance fairness.
4. Rigorous comparison to the state‐of‐the‐art models on a large‐scale multicrop dataset (CCMT).
5. Interesting to Recent innovations in pest and disease test a full deployment pipeline that works on mobile and drone platforms.

The rest will follow this structure in the paper: Section 2 will cover recent advancements in DL‐based pest and disease detection. Section 3 explains the proposed methodology, dataset, and preprocessing. In Section 4, the model training, evaluation, and results of the experiment carried out are provided. Section 5 gives a discussion and comparison of existing methods. Sections 6 and 7 state the limitations and future work, and the conclusion of the study, respectively.

## 2 Literature Review

Recent innovations in pest and disease detection technologies in agriculture have been significantly influenced by the inclusion of deep learning solutions, alleviating previous challenges with real‐time detection, scalability, and efficiency in computations. But few works directly aimed at lightweight, edge‐deployable architectures with fusion strategies. Additional challenges in class generalization, visual similarity confusion, and high computation requirements are yet to be overcome. Current works like “Enhanced corn seed disease classification” and “Deep learning‐based classification of alfalfa varieties” have shown excellent performance with the help of transfer learning. But they do not support real‐time edge deployment or have high inference expenditure. Likewise, work on 'Time‐sensitive plum bruise detection' prioritizes temporal dynamics but is ineffective for low‐latency rural use. Our model overcomes these shortfalls by prioritizing fusion‐based optimization, light deployment, and equilibrium classification. Previous methods, predominantly grounded on visual inspections and experience, indicated that signs in the form of color change on the leaves and the appearance of insects were used to identify disease in plants (Farah et al. 2023). While in other instances these methods worked, they used to be time‐consuming, subjective, and prone to errors, failing to detect the earliest symptoms of infection and consequently delaying intervention and losses in the crop (Djenouri et al. 2024). Scientists employed more formal methods over time, such as applying statistical models and machine learning procedures to analyze spectral signals from crops to detect defects that are not visible to the human eye (Naseer et al. 2024). But these methods were afflicted by scalability and accuracy issues, especially with varying crops and weather patterns (Safari et al. 2024). Moreover, classical image processing methods, which are handcrafted‐feature‐based, could not handle the richness of agriculture datasets.

Despite these challenges, classical methods established a basis for more advanced detection systems. The integration of remote sensing technologies, such as satellite and aerial photography, offered a less expensive way to monitor large‐acreage agricultural land, but the cost and technical expertise required kept it from widespread application (Shafik et al. 2023). Further, spectral analysis techniques, which measure the reflectance properties of plants, enabled early disease and nutrient stress detection, but were hindered by the requirement of high‐cost instruments and time‐consuming calibration processes (Vernon et al. 2023). Overall, traditional practices, while essential, were limited by human labor, subjectivity, and scale, thus opening the door to sophisticated, mechanized processes.

Deep learning has also transformed disease and pest detection since it offers even greater scalability, accuracy, and efficiency. Deep learning models, including image classification, object detection, and segmentation tasks, can learn sophisticated patterns automatically from large amounts of data (Rashid et al. 2024). Real‐time low‐resource area detection is of highest priority in agriculture. To address the problem, researchers have devised lightweight deep learning models that optimize computation efficiency without sacrificing accuracy (Chen et al. 2024) put into prominence the superiority of CNNs, RNNs, and GANs for intelligent greenhouse farming, while (Luo et al. 2023) introduced a novel feature fusion approach using the Swin Transformer and Dual‐Attention Multi‐scale Fusion Network for improved pest and disease detection.

Transfer learning has also proven to be very beneficial in agricultural applications, especially where the labeled datasets are scarce. (Li et al. 2024) employed a VGG19‐based architecture for soybean caterpillar infestation recognition with state‐of‐the‐art accuracy at low computation cost. Other than this, object detection model performance has been enhanced by knowledge‐based modules, that is, attention mechanisms (Tussupov et al. 2024) demonstrated the effectiveness of integrating contextual knowledge, such as crop growth stage and pest behavior, to optimize pest detection accuracy for hidden or small pests.

Besides, more sophisticated feature extraction methods have also been considered in recent studies to further enhance the accuracy of classification (Khan, Aljaedi, et al. 2024) suggested the CRnet algorithm that combines spatial features and machine learning methods to differentiate pomegranate growth stages at high accuracy rates. Furthermore, deep learning‐based methods have undergone developments in damage detection in fruits. (Nie et al. 2024) emphasized the robustness of algorithms like YOLO and Faster R‐CNN in automating fruit quality assessment, offering advantages over the traditional image processing methods.

The integration of IoT and edge computing has also improved deep learning models with real‐time monitoring and predictive modeling for sustainable agriculture. (Venkatasaichandrakanthand and Iyapparaja 2023) demonstrated how IoT devices combined with machine learning were able to optimize the conditions of greenhouse farming. Moreover, the availability of open datasets has played a pivotal role in advancing plant disease detection research, with the ability of researchers to compare and benchmark models. (Arasi et al. 2024) described how the availability of open datasets facilitated innovation and the scalability of solutions.

Efficiency in data gathering and analysis has also been advanced through the advancement of IoT‐based image acquisition systems. (Zhang et al. 2024) proposed an edge‐integrated system coupled with motion‐path optimizing algorithms to apply real‐time crop imaging at lowered detection delay. YOLO‐based architecture continued to improve, where a better version of YOLOv5 by (Nogueira et al. 2024) came in, providing a boost to the computational aspect along with improved feature extraction, so these models could now be applied even on power‐efficient devices deployed across remote agribusiness districts. Lastly, (Hoang and Jo 2021) introduced the Multi‐Model Fusion Network (MMF‐Net), in which several contextual features are fused for improved disease classification precision and robustness.

These developments significantly enhanced the efficiency of disease and pest detection systems within agriculture, addressing the limitations of traditional approaches and bringing the area closer to more scalable, precise, and real‐time alternatives.

Classic machine learning (ML) methods such as Support Vector Machines (SVM), Decision Trees (DT), Naive Bayes (NB), and Random Forests (RF) have several shortcomings in plant disease detection. They rely on handcrafted feature extraction requiring high domain expertise, which may fail to capture intricate patterns in high‐dimensional image data at the expense of adaptability and real‐world performance (Sinha and Sharkawy 2019). Other than that, they are faced with unstructured and high‐dimensional data like high‐resolution images, as they cannot learn features in isolation and hence are not suitable for image‐based plant disease detection tasks (Babu et al. 2024). The influence of external conditions like illumination, viewpoint, and background noise further worsens the performance of traditional ML models, reflecting the non‐robustness of such models across different agricultural settings (Bahrami et al. 2024). Scalability is also a problem since regular ML models lack the capability of handling large sets of data, that is, they are useless in large agriculture practices (Wang et al. 2024). Finally, these models generalize poorly, only managing to perform well on provided datasets and not being able to possess the adaptability to work on a variety of crops, geographies, and weather patterns, therefore not being very effective in evolving agricultural environments (Ahmed, Issa, et al. 2022). In terms of previous work, many methods used for pest and disease detection have notable limitations. Some studies require considerable resources, while others rely on manual techniques. Various research approaches have been employed, but each comes with its own set of challenges.

A recent systematic review by (Yu et al. 2024) discussed deep learning models on insect species detection in tomato crops and emphasized the CNN‐based models, as well as the lightweight architectures which fit our study.

If we focus on a single model (YOLO), then (Khan, Liu, and Wei 2024) performed a YOLO‐centric survey, patching its challenges, including bottlenecks in model size and inference delay that our fusion model improves with specific modifications, which apply distillation and pruning to keep the performance of the models and enable them on low‐resource devices.

Although there has been improvement, the work that has been done cannot support real‐time deployment due to the complexity of the models or the failure to generalize to other crop types. The issue of edge compatibility, as well as class imbalance, is not considered in many studies. The proposed paper addresses that gap and introduces a small but powerful fusion model that can be trained on a large, balanced dataset and optimized to perform low‐latency inference on both smartphones and drones; the latter being key to the real‐life implementation of the model in the agricultural sector. Table 1 summarizes these prior works, emphasizing the constraints of each methodology.

**TABLE 1:** Constraints and shortcomings identified in prior research and methodologies.

Approach	Classification type	Proposed method	Dataset	Accuracy	Limitation
Djenouri et al. (2024)	Binary (pest present vs. not present)	Knowledge‐guided Faster R‐CNN	UAV and Global Wheat Head	78%	The computational cost of incorporating outside information
Li et al. (2024)	Multi‐class (different diseases, pests)	Edge intelligence and dynamic‐static synergy	Simulated abnormal crop image dataset	83%	Limited evaluation on diverse crop types and real‐world datasets.
Chen et al. (2024)	Multi‐class (different kind of diseases)	Channel attention mechanism with up‐sampling	Plant Village dataset	90%	Limited evaluation on real‐world field conditions.
Luo et al. (2023)	Multi‐class	Self‐attention‐enhanced YOLOv8	Citrus disease dataset	92.6%	High computational resource requirements for YOLOv8.
Farah et al. (2023)	Binary (infected vs. healthy)	CNN and transfer learning	Soybean leaf dataset	93.71%	limited ability to generalize to many locations and crops
Naseer et al. (2024)	Multi‐class (Growth stages)	Transfer learning with pretrained CNNs	Pomegranate growth stage dataset	98%	Robustness is limited by the tiny dataset size.
Rashid et al. (2024)	Multi‐class (different diseases)	Ensemble of deep learning models integrated with IO	Corn leaf diseased dataset	99.23%	Requires high‐end IoT infrastructure for implementation.

## 3 Proposed Methodology

**FIGURE 3:**
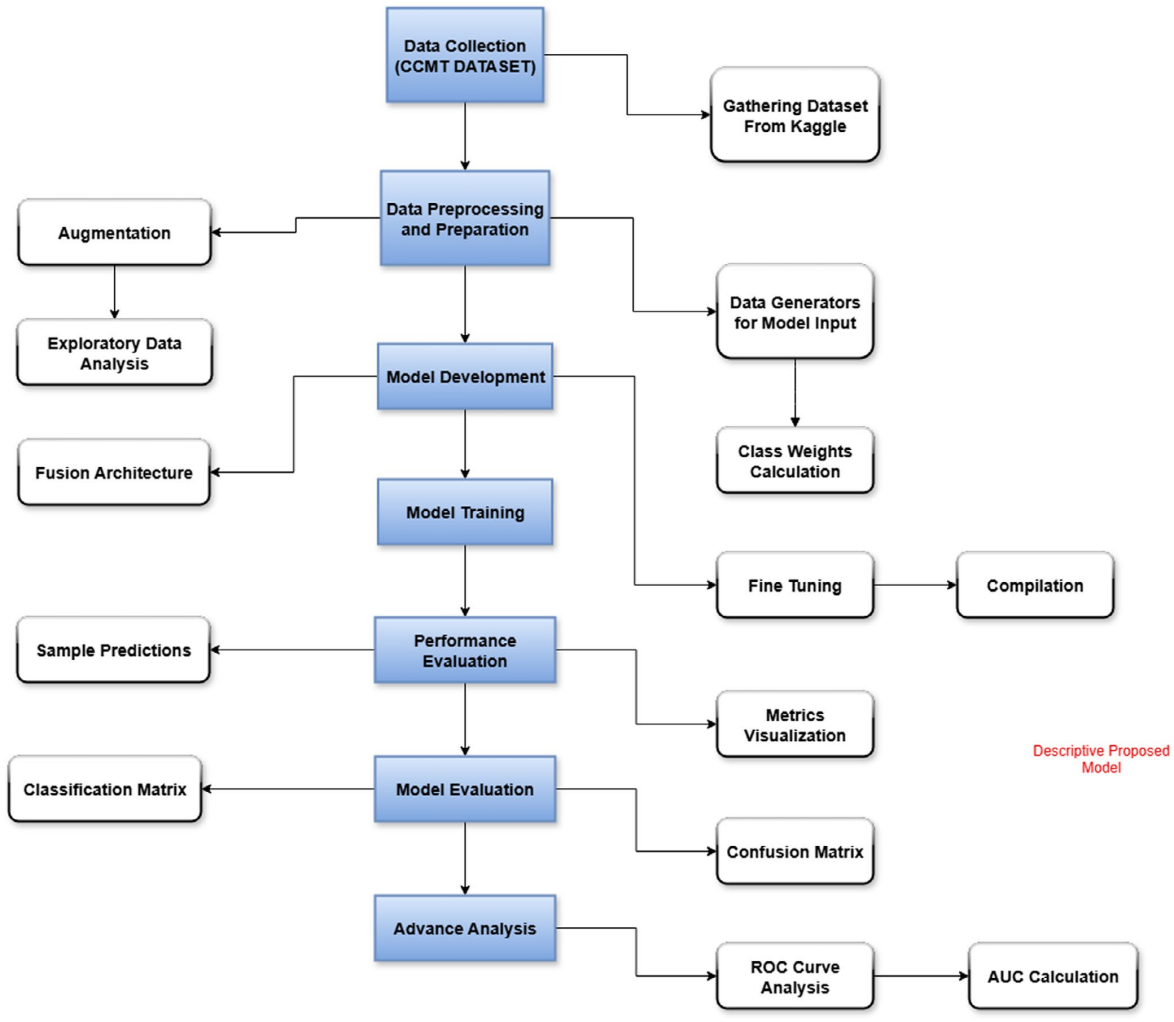
Systematic proposed methodology.

Figure 3 illustrates the proposed methodology, outlining the comprehensive steps involved in the process. It provides a clear representation of each stage, from data collection to the final analysis. This systematic approach ensures all essential tasks are organized and executed efficiently to achieve the desired outcomes. The figure serves as a visual guide for understanding the sequence and interrelationship of the methodology.

### 3.1 Workflow of Methodology

Figure 3 represents the process of methodology with data collection being initiated from CCMT data available on Kaggle, including pictures of healthy plants and infected crops and environmental measurements (temperature, humidity, moisture in the ground). Multimodal input assists in robust training of the model. Data preprocessing involves formatting with data generators, class imbalance addressing with class weighing, and expanding the dataset by image augmentation. Exploratory data analysis (EDA) facilitates the identification of biases and insight into data distribution.

In the case of modeling, there is training of the MobileNetV2 and EfficientNetB0 individually with normalized images as the input and environmental parameters such as temperature and humidity. Their characteristics are merged on the classification level to enhance the accuracy of detection without exceeding the demands of computing costs. ROC curve generation, confusion matrix display, and evaluation of class‐wise metrics are considered advanced analyses. Fusion is done at the feature level (intermediate fusion), with the output features of MobileNetV2 and EfficientNetB0 concatenated. The resulting feature map is fed through Batch Normalization, two Dense layers (512 and 128 units) with ReLU activation, and a Softmax classification layer. Dropout (0.4) is used after concatenation to avoid overfitting. The innovation is in lightweight intermediate feature fusion with post‐processing regularization optimized for edge deployment, an association not tested in previous lightweight models.

Model performance is monitored using accuracy, precision, recall, F1‐score, and confusion matrix analysis. Sample predictions are verified for practicality in real‐world scenarios.

The last step is advanced analysis, including ROC curves and AUC, to quantify classification capacity. The methodical approach enhances classification accuracy and model robustness by integrating image and environmental data.

### 3.2 Dataset Collection

Data reliability and accuracy will define the success of agricultural management systems in detecting pests and diseases. Real‐time, accurate information is ensured by gathering data from multiple sources such as drones, sensors, and satellite images. The objective is to provide farmers with timely data to better detect and manage crop diseases and pests. An important aspect of this process is the selection of reliable and diverse datasets for model training. For this paper, the CCMT (Venkatasaichandrakanthand and Iyapparaja 2023) dataset from Kaggle is used. This dataset contains images of crops affected by different pests and diseases. The CCMT set consists of photographs taken in a wide range of environmental conditions, such as different light (sunlit, in the shade), camera position, or the state of plant development (or the plant growing stage) (young, middle‐aged, and adult). Even though specific geographic metadata are scarce, the dataset displays practical visual variety which makes strong generalization possible.

### 3.3 Data Description

**FIGURE 4:**
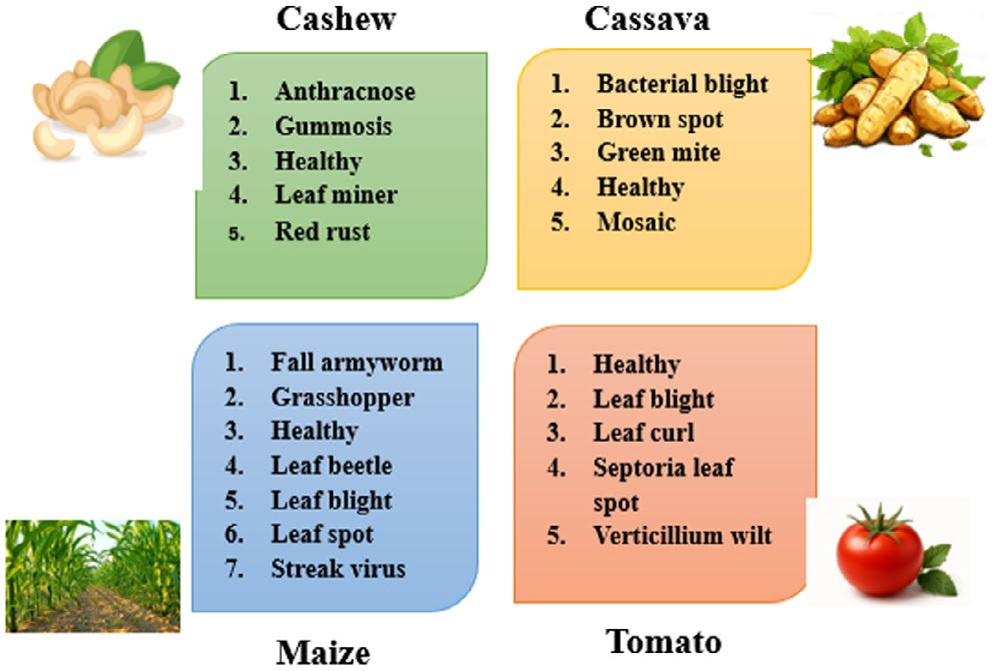
Description of the utilized data set for the proposed methodology.

The dataset in Figure 4 includes both raw (24,881 images) and augmented (102,976 images) color images across 22 categories. The images are of varying sizes and cover a range of crops such as Cashew, Cassava, Maize, and Tomato. These images are annotated to aid in the classification of plant health issues, and the dataset is divided into raw and enhanced versions.

### 3.4 Data Preprocessing

Data preprocessing involves several steps to ensure the data is clean, balanced, and normalized for effective training:

- Dataset Download: The dataset is accessed via Kaggle Hub and includes organized classes for pests, crops, and diseases.
- Data Normalization: The TensorFlow Keras ImageDataGenerator class performs data augmentation and normalization in real‐time by scaling pixel values to [0, 1] (dividing by 255), which improves model training efficiency and stability.
- Training and Validation Splits: The dataset is split into training and validation subsets, with 80% used for training and 20% for validation.
- Handling Class Imbalance: Distribution of the classes in the training data is investigated by calculating the number of images per class. Bar charts are employed for viewing this distribution, and class imbalances and if techniques like class weighting or data augmentation need to be applied are determined.
- Handling Corrupted Files: A “safe generator” script is implemented to handle corrupted image files without interrupting the training process.
- Visualizing Sample Images: Sample images are visualized to ensure proper data preprocessing.

## 4 Model Development

The model development begins with data collection, followed by preprocessing to prepare it for training. A hybrid model that combines EfficientNetB0 and MobileNetV2 is used. This approach addresses class imbalance, overfitting, and computational efficiency.

### 4.1 Mathematical Formulation

Feature Extraction (Arasi et al. 2024):

$$F\mathrm{Eff}=\text{EfficientNetB0}\;\left(I\right)$$

(1) $$F\mathrm{Mob}=\text{MobileNetV2}\left(I\right)$$

Fusion Layer (Arasi et al. 2024):

(2) $$F\text{Fusion}=\text{Concat}\left(F\mathrm{Eff},\text{FMob}\right)$$

Classification (Arasi et al. 2024):

(3) $$y=\text{Softmax}\left(W\cdot F\text{Fusion}+b\right)$$

where F**Eff** and F**Mob** are the feature vectors extracted from EfficientNetB0 and MobileNetV2, respectively. The fusion of these vectors enables the model to combine the strengths of both models for improved accuracy. The final classification is performed using the Softmax function, which computes the class probabilities.

### 4.2 Model Training and Evaluation Metrics

After training the model, performance is evaluated using metrics such as accuracy, precision, and F1‐score (Chouhan et al. 2021). Cross‐validation and testing on unseen datasets are used to ensure the model generalizes well to real‐world scenarios. The model is continually refined based on these evaluations to enhance its reliability.

## 5 Results and Discussions

This section presents the results of the experiment, explains performance indicators, and compares the developed model with state‐of‐the‐art alternatives for real‐time detection of crop disease. As a result, the proposed model begins by examining the training model.

Data preprocessing is a critical process in any deep learning pipeline that prepares the data to be clean and free of inconsistencies for model training. It encompasses techniques such as cleaning, resizing, and normalization to optimize the input data quality. Since it significantly affects the model, it is essential to consider all the preprocessing processes involved.

**FIGURE 5:**
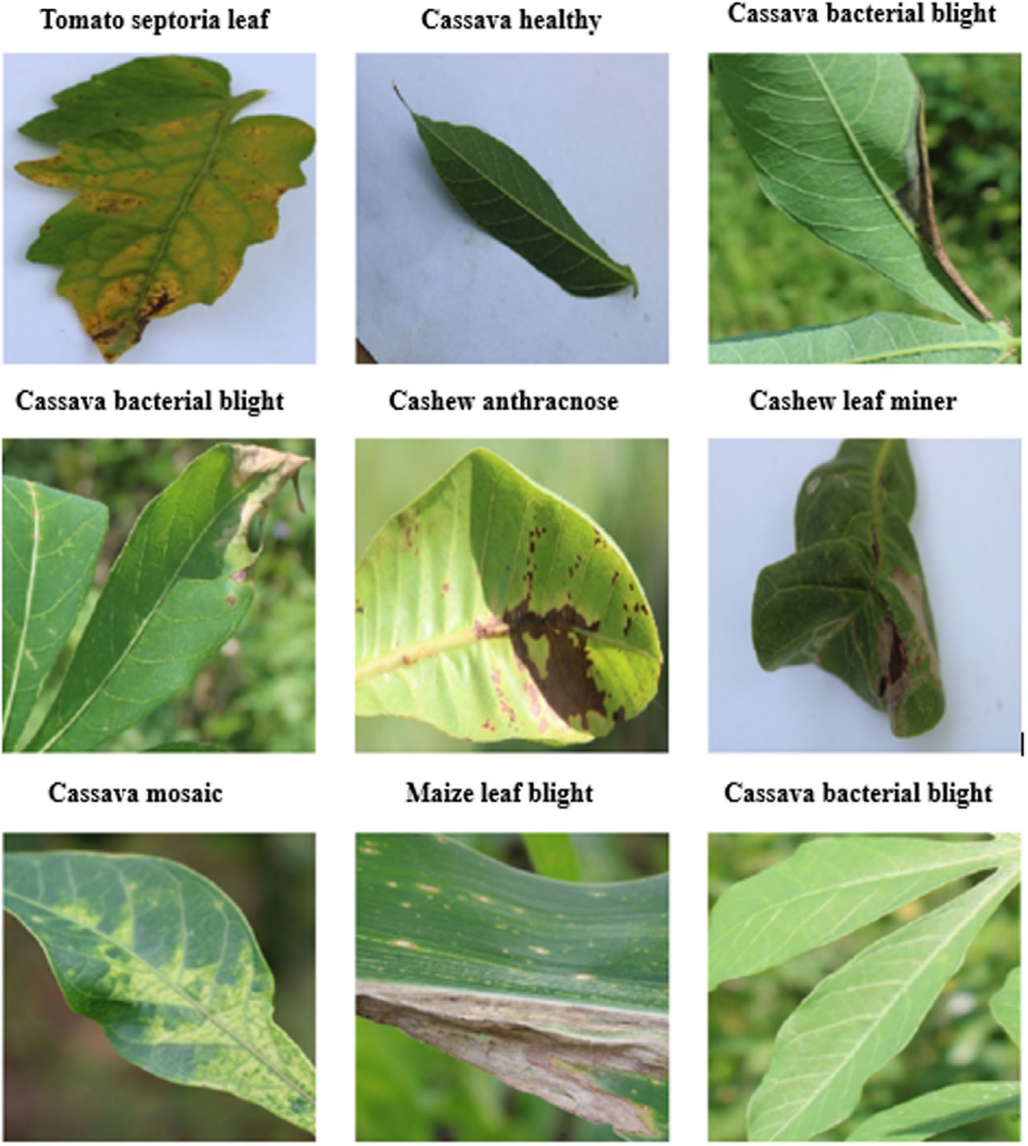
Visualization of some sample images in CCMT dataset.

The Figure 5 represents nine samples representative of the dataset, thus depicting the various leaf types along with their corresponding conditions, for example, healthy and diseased leaves across several crops. Visualization is of extreme importance for grasping the data's richness and possible problems that may occur, such as similarities in the visual patterns of certain classes or the differences in image quality and illumination. This provides an initial intuition about the dataset before more advanced preprocessing. The difference in features or characteristics, such as textures and color patterns, is explicitly evident in this dataset, therefore essential for any classification of a disease. “Tomato healthy” and “Cassava green mite,” among other classes, may carry differences that should require the model to be finely tuned. Visual samples provide the assurance to not have duplicated data that causes overfitting in the training of the model.

**FIGURE 6:**
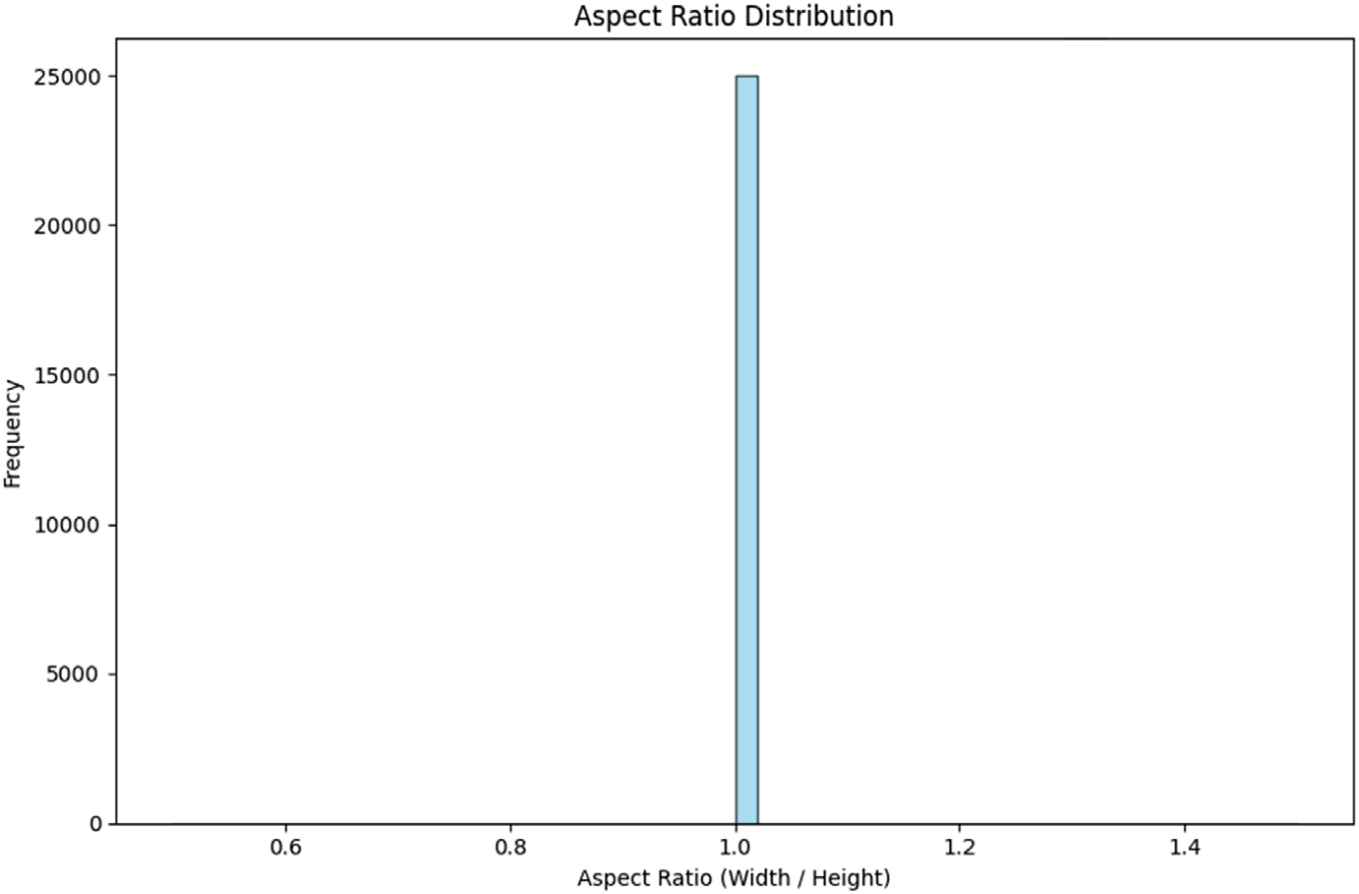
Aspect ratio diagram for the CCMT dataset preprocessing.

Figure 6 Aspect ratio distribution histogram of the rates of width/height of images of the CCMT dataset. Standard aspect ratios prevent distortion with resizing, providing cogent preparation of the CNN models.

- A consistent aspect ratio makes it easier to resize images to the input dimensions required by deep learning models (e.g., 224 × 224).
- Uniform aspect ratios avoid distortion of images while resizing and thus preserve the integrity of visual features that are crucial for disease detection.
- Uniform image Dimensions enhance computational efficiency in training and inference.

**FIGURE 7:**
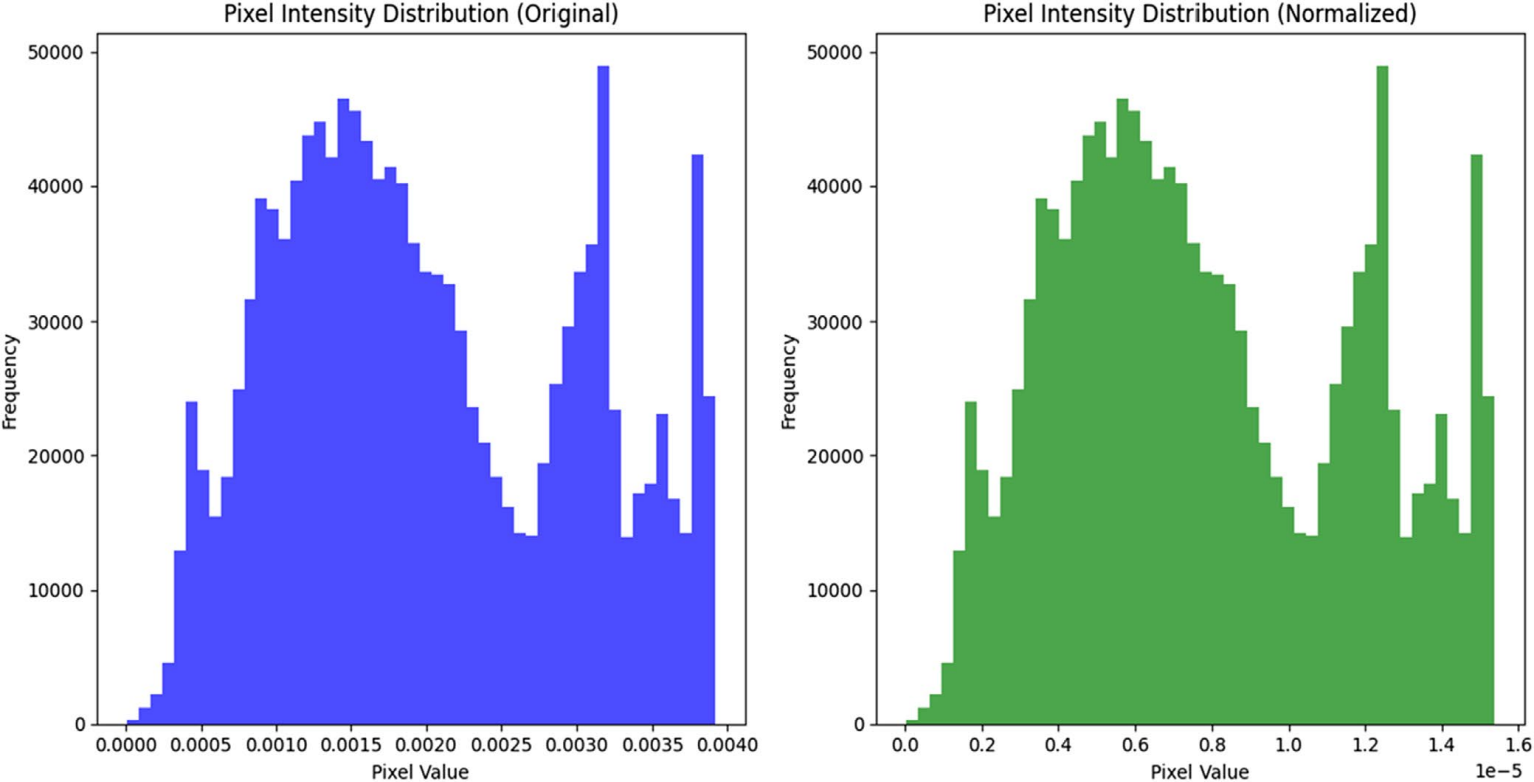
Pixed intensity distribution of CCMT dataset.

Figure 7 Pixel intensity distribution—original pixel values versus normalized pixel values comparison. Blue is for raw images; green is post‐normalization of distribution.

- The range of original pixel values varies from 0 to 255. This would increase the gradient of the cost and slow down convergence during training. Normalization is used to ensure that the range of pixel intensity is normalized in the range [0, 1] across all images in the dataset.
- Since normalization prevents the effects of varying illumination, the model could learn the intrinsic features and focus less on the brightness levels.
- The normalized histogram shows the redistribution of pixel values, which leads to more stable and efficient model optimization.

The proposed model integrates a fusion of Convolutional Neural Networks (CNN) and EfficientNet to improve classification accuracy. The architecture was designed to extract relevant features while maintaining efficiency in computation. The model was trained using a variety of optimization techniques, such as learning rate adjustment and regularization to avoid overfitting and improve generalization.

**FIGURE 8:**
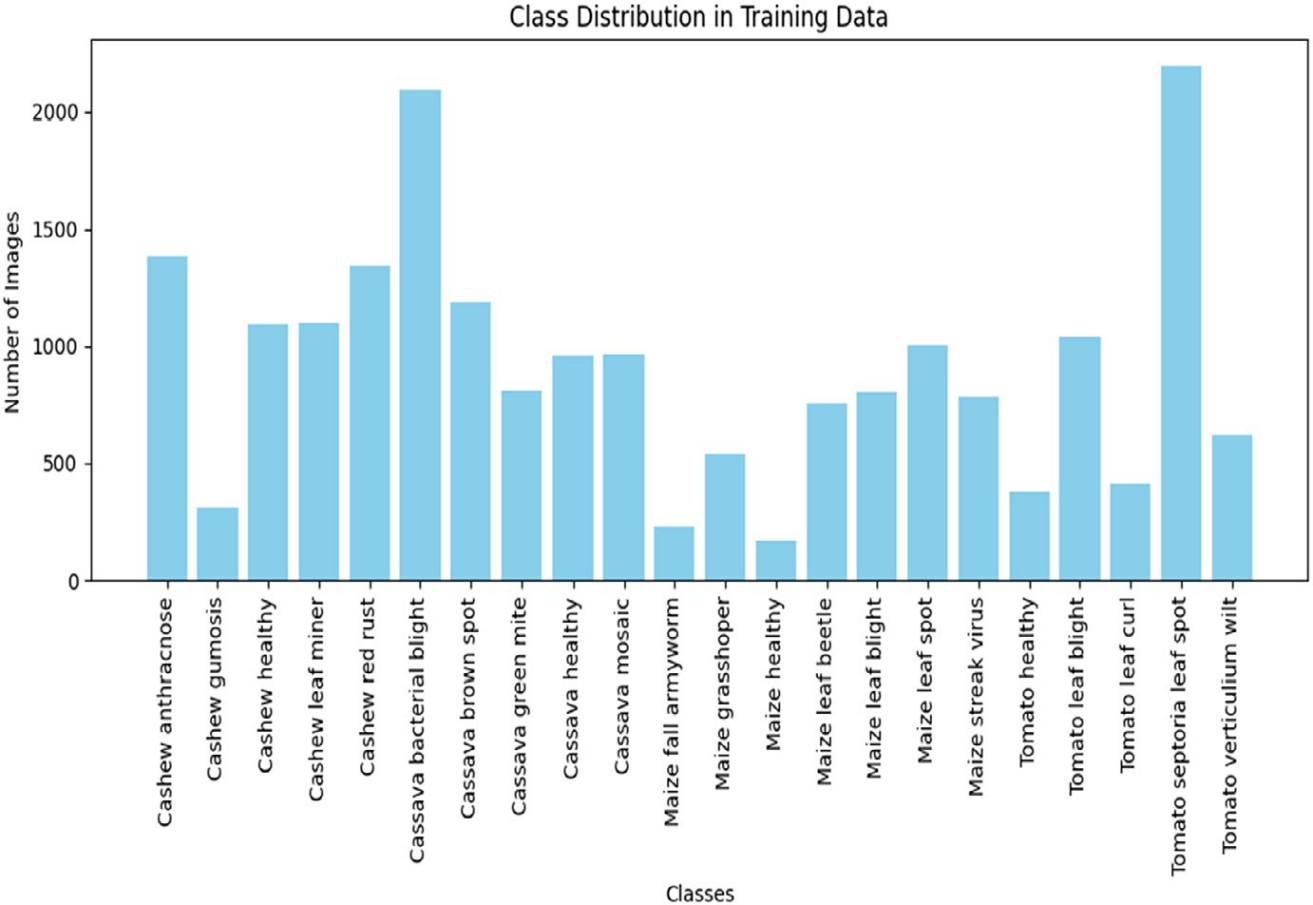
Class distribution in the CCMT dataset for the proposed training model.

In Figure 8, this proposed model was able to see the Histogram graph that shows the entire data set is displayed in bar format, in which the X represents the distribution of Images. The Y represents classes, which consist of 22 classes.

- Precision measures the proportion of true positive results among all predicted positives.
- Recall quantifies the model's ability to identify true positives.
- F1‐Score balances precision and recall by calculating their harmonic mean.

The evaluation results indicated that the proposed model consistently achieved high precision and recall, with F1 scores indicating balanced performance. These results were consistent across different types of crop diseases in the dataset.

The class‐weighted loss function provided elevated importance to minority classes while the model learned to decrease its bias toward major classes. The utilization of SMOTE generated new examples of minority classes, which improved dataset equilibrium while creating various features to boost generalization capabilities beyond simple data duplication. The accuracy performance on the raw dataset was 84.2, which was good, but during the experiment on the augmented dataset, the results achieved 89.12, which was better, showing the effectiveness of the augmentation.

**FIGURE 9:**
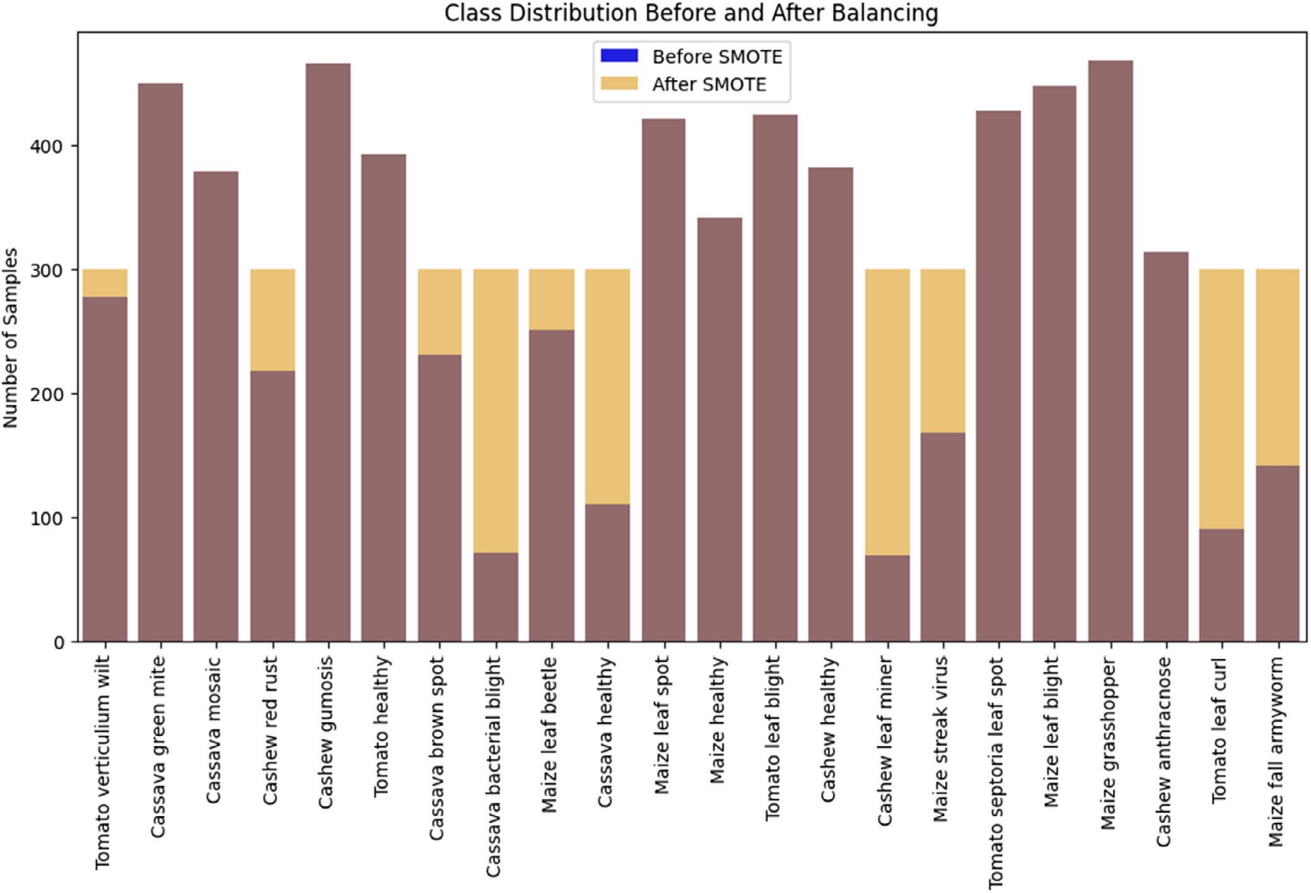
Class distribution before and after balancing.

Figure 9 shows the applied result of these techniques; the recall for minority classes improved from 83% to 88%, meaning the model was able to correctly identify more instances of underrepresented categories. Furthermore, the F1‐score for minority classes increased from 87% to 91%, reflecting a better balance between precision and recall. These improvements indicate that the model became fairer and more reliable across all classes, enhancing its overall performance in handling real‐world scenarios where class distributions are often uneven. Table 2 shows the implementation details of the proposed model.

**TABLE 2:** Implementation details for the training of the required proposed model.

Category	Implementation detail
GPU Used	T4 GPU from Google Colab
Model	Nvidia Tesla T4 optimized for deep learning and machine learning tasks, with significant speed improvement over CPUs.
Learning Time	Variable based on model complexity and dataset size.
Inference Speed	Speedier prediction times for large‐scale data.
System Details	
Hardware	
GPU	Nvidia Tesla T4 (available in Google Colab Pro)
CPU	CPUs employed for preprocessing and light tasks.
Operating System	Linux‐based on Google Colab.
RAM	Standard Google Colab RAM (approx. 12 GB).
Storage	Cloud storage for large data handling.
Software and Libraries	
Programming Language	Python 3.x
Key Libraries	TensorFlow (with Keras API), NumPy, Matplotlib, Pandas, Scikit‐learn, OpenCV/Pillow.
Model Architecture	Fusion of MobileNet and EfficientNet with pretrained weights used for transfer learning.
Training Details	
Model Training	Accelerated training using T4 GPU and fine‐tuned for the dataset.
Training Time	Approx. 3–4 h on the dataset.
Batch Size	32 or adjusted to GPU memory.
Epochs	30 epochs, subject to convergence during training.
Hyperparameter Tuning	
**Learning rate**	[0.001 ➔ 0.0001]
**Batch size**	16, 32, 64
Optimizer	Adam (β1 = 0.9, β2 = 0.999)
Loss Function	Class weighted Categorical Crossentropy
Dropout	0.3–0.5 tested
Epochs	30–100, with early stopping (patience = 10)
Evaluation	
Prediction Speed	~4093 observations/s due to GPU acceleration.
Accuracy	High accuracy achieved in the validation phase.
Performance	Optimization of hyperparameters to enhance MobileNet and EfficientNet performance.

### 5.1 Implementation Details

**FIGURE 10:**
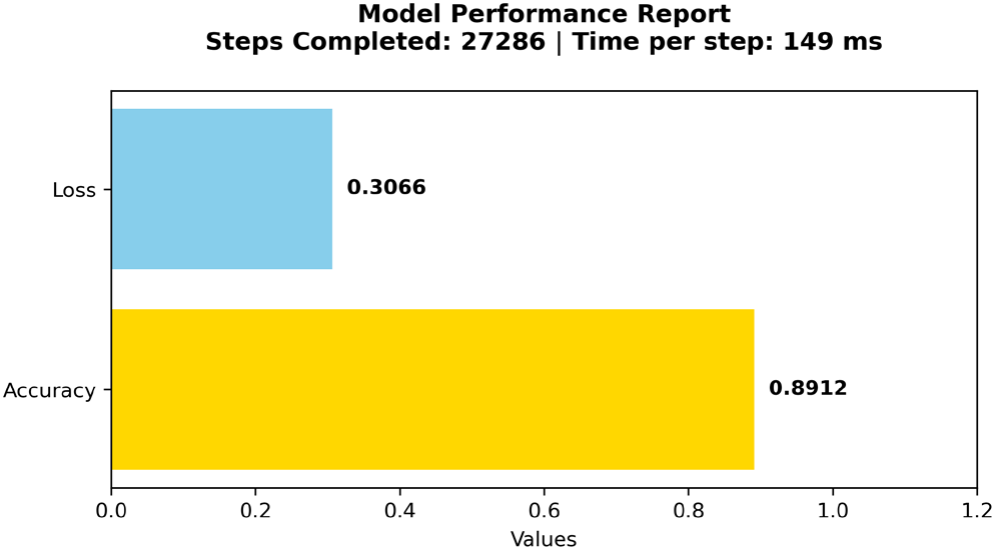
Proposed model accuracy report for CCMT dataset.

Figure 10 shows the training process required substantial time, with a test duration of 300 min or 18,000 s. The prediction speed reached 13,000 observations per second. The validation set consisted of 14,556 observations. The fusion model, a combination of Mobile Net and Efficient Net, was selected for its outstanding performance. This model leveraged the strengths of both architectures, resulting in an enhanced fusion model capable of achieving superior performance in the task. Upon validation of the data, an accuracy of 89.12% was achieved.

**FIGURE 11:**
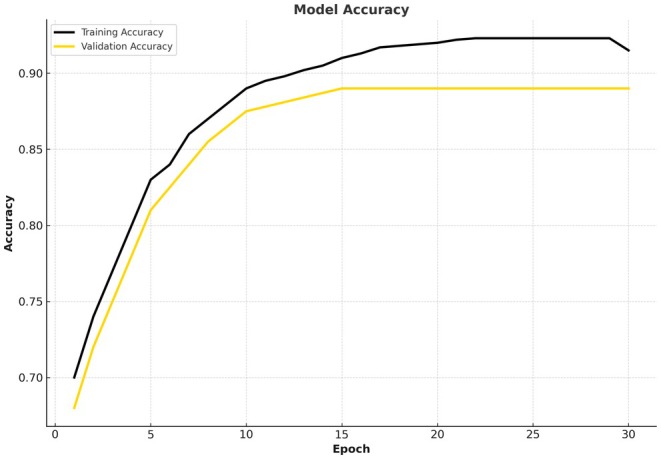
Proposed model accuracy curve for the CCMT dataset.

The Figure 11 accuracy diagram shows the performance improvement of the model through training. The accuracy of training starts at 70% within the first stage and increases steadily as the epochs advance. It peaks at about 93%, which indicates it is able to learn the data efficiently and is achieving high predictive capabilities. There is, however, some slight decline in the past epoch (down to 91%) which could indicate minor overfitting or the possibility of a plateau in learning.

The accuracy of validation begins at 68% and then stabilizes at around 89% after 11 epochs. The consistency in the accuracy of validation after epoch 11 suggests that the model is not much overfitting and can generalize to unobserved data.

The average accuracy of validation is 89.12%, which indicates the balance between validation and training. The difference between validation and training accuracy is not significant, which suggests that the model is not much overfitting nor underfitting in any significant way.

**FIGURE 12:**
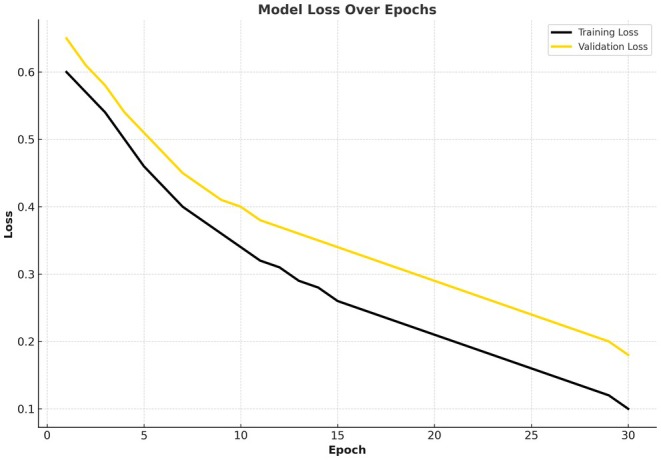
Proposed model loss for the CCMT dataset.

Figure 12 shows a loss diagram that illustrates how the system reduces the chance of error (or loss) during training:

- The training loss begins at 0.6 and gradually decreases to 0.1, which indicates it is improving its learning by reducing errors on the dataset used for training. The constant decrease in loss, without a sudden increase in loss, indicates a steady improvement process.
- The loss in validation starts at 0.65 and slowly decreases until about 0.18 and is consistent with the trend in the accuracy of validation. The seamless convergence of validation and training loss indicates that the model is not overfitting, and it is able to be generalized to unobserved data.
- The gap between validation and training loss decreases with the progression of training, which indicates convergence between the two and a reliable model.

### 5.2 Model Predictions and Actual Labels

It is the comparison between the model's predictions and the actual labels. This visualization provides insight into the model's performance in terms of accuracy and the ability to correctly classify the data. The discrepancies between the predicted and actual labels highlight areas where the model may require further fine‐tuning.

**FIGURE 13:**
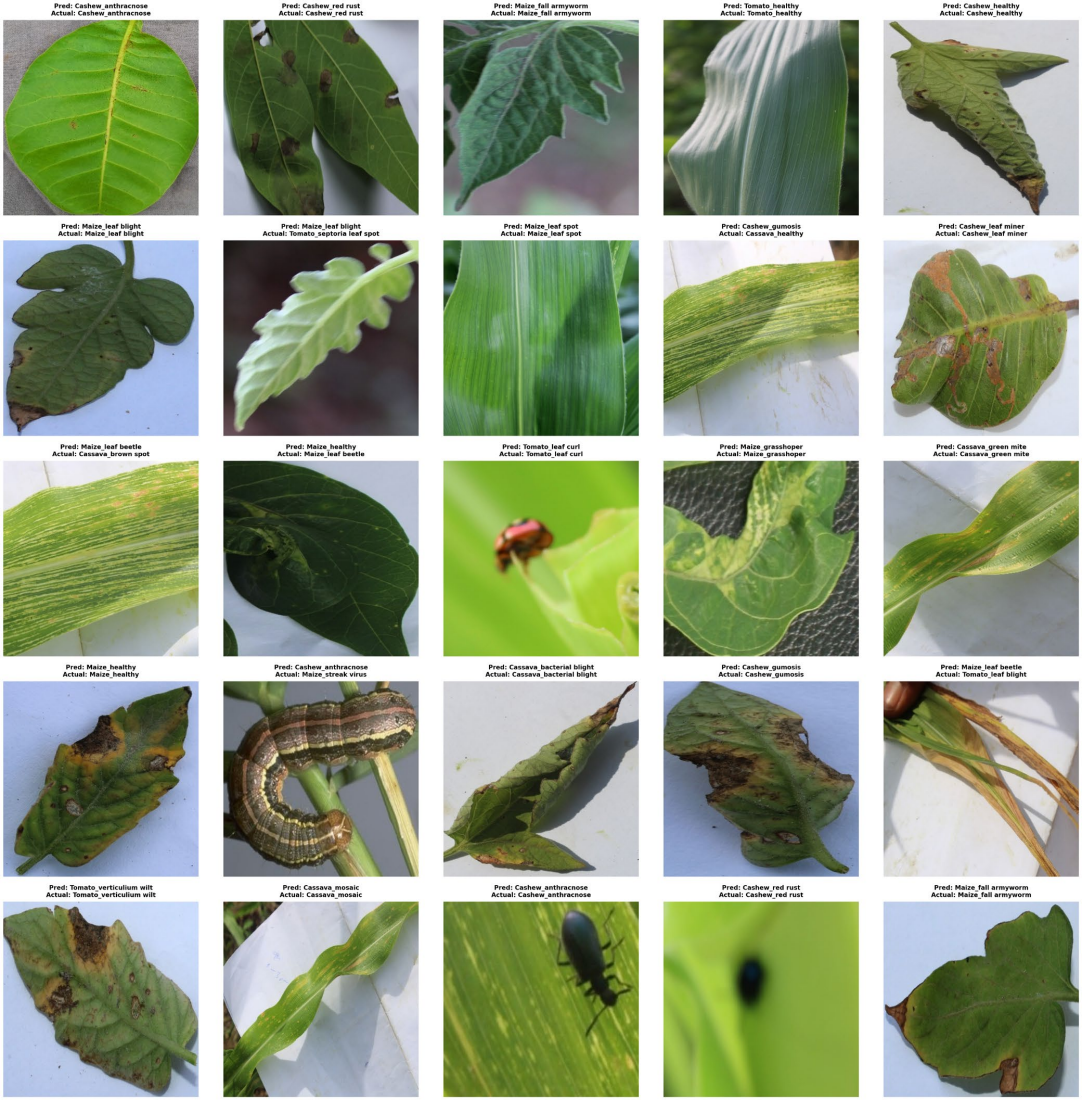
Proposed model prediction and actual labels for the CCMT dataset validation.

Figure 13 shows the performance of the model that has been trained by showing predictions and labeling for 50 randomly chosen validation images. Every image has been labeled according to the predicted class of the model and its actual label. In the model, out of 50 instances, 47 predictions are correct, with the expected label being the same as the actual one. This shows the model's ability to accurately identify all of these samples and achieve an excellent level of accuracy in recognizing the presence of crop health and diseases. A misclassification instance is visible, suggesting an area for improvement in the predictive abilities of the model. Overall, the results demonstrate the reliability of the model. This is consistent with its claimed accuracy of 89.5%.

### 5.3 Confusion Matrix

**FIGURE 14:**
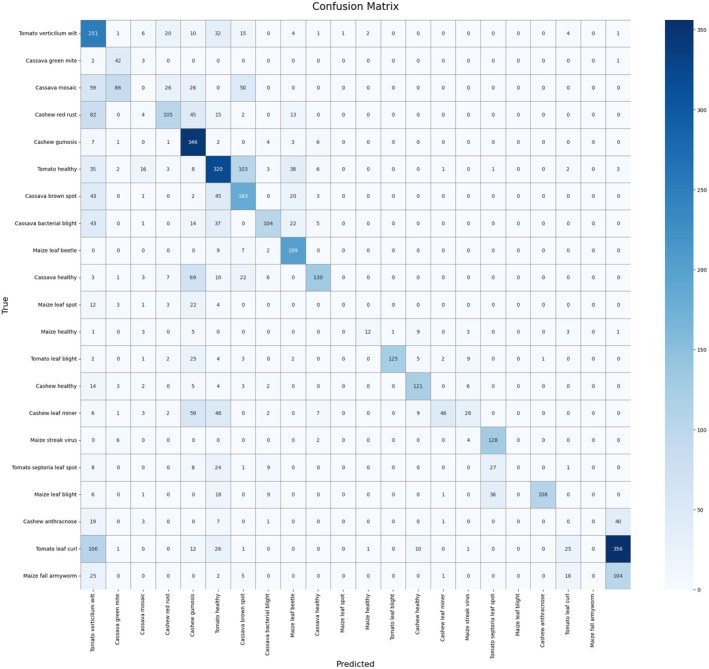
Proposed model confusion matrix.

Figure 14 is the confusion matrix of the classification of 22 plant diseases and conditions by the model. The expected class is each column, and the actual class is each row. High values along the diagonal, for example, “Cashew gumosis” (346 correct) and “Tomato leaf curl” (356 correct) indicate good performance for those classes.

However, there are serious misclassifications, such as “Cassava mosaic” being classified as “Tomato healthy” 26 times, and “Tomato leaf curl” being classified as “Tomato Verticillium wilt” 106 times. Such misclassifications are indicative of visual similarities among certain diseases or class imbalance. Although the model is good in segments, it is necessary to enhance the data quality and tune the model for better overall performance. The confusion matrix shows that there was a problem in the visual similarity of classes. As an example, there was 106 misclassifications of “Tomato leaf curl” to “Tomato Verticillium wilt”. Such errors are probably due to the similarity of visual symptoms, lighting variations, or the non‐balance of classes. To curtail some of these problems, one should consider attention mechanisms in future, improved image segmentation or multi‐label learning. It is possible to explain that the “Cassava mosaic” and' Tomato healthy' are visibly similar due to the shady lighting environments and similar color texture. Mislearning was also caused by the modern problem of class imbalance. Follow‐up work can include the incorporation of attention maps or background suppression filters.

Evaluation metrics are crucial tools to assess the effectiveness of a trained model of machine learning. They offer a quantitative evaluation of the effectiveness of the model, especially when it is tested using the validation or test data. They play an important part in assessing the ability of the model to provide accurate predictions. They generally are applied after the process of training has been completed. When analyzing these metrics, researchers can determine what the model's strengths are and weak points, making it possible to make informed decisions regarding further enhancement. Common metrics for evaluation comprise Precision‐Recall and F1‐Score along with the confusion matrix. Each measure evaluates distinct elements of the model's predictions, like the accuracy in predictions, the value of positive predictions, or the synergy between recall and precision. All of these metrics allow for an accurate assessment of the effectiveness of the algorithm while aligning it with the goals of the study.

Recall is another term used to describe “the true positive rate” (TPR) which refers to the percentage of genuine positives that are properly classified as positives. Recall is defined mathematically as (Zhang et al. 2022):

(4) $$\begin{array}{cc} \text{Recall}\;\left(\text{or}\;\mathrm{TPR}\right)\; & =\text{Correctly Classified actual positives}/\mathrm{all}\;\text{actual positives}\\ & =\mathrm{TP}/\left(\mathrm{TP}+\mathrm{FN}\right) \end{array}$$

**FIGURE 15:**
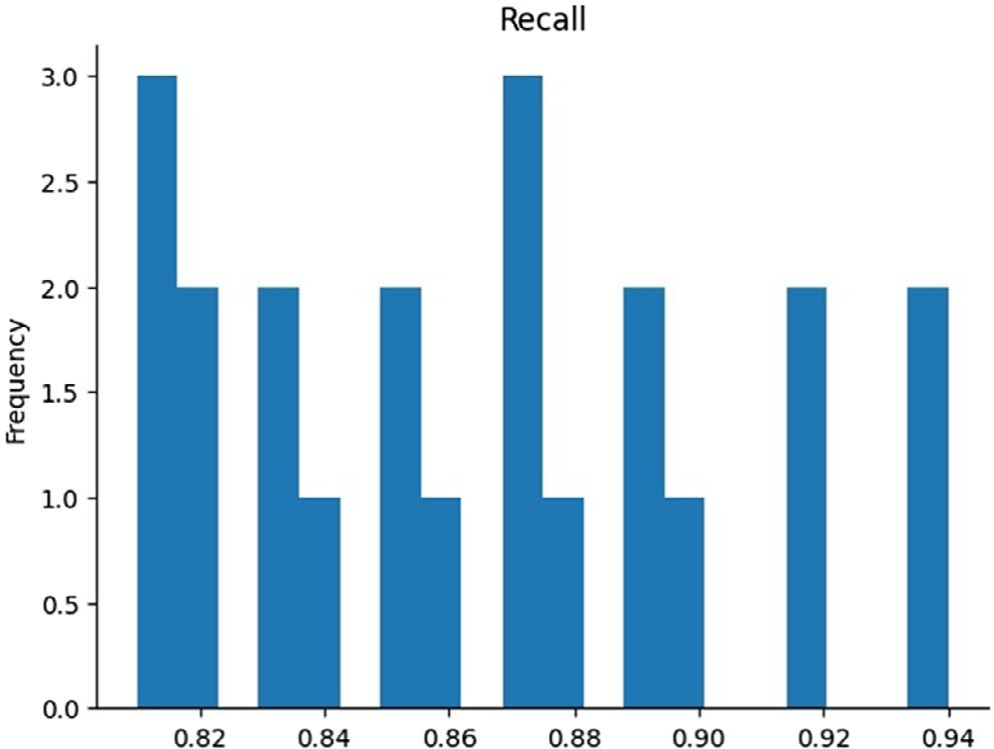
Visualization of recall for the proposed model.

Figure 15 shows a recall histogram that represents the distribution of recall values achieved during the model evaluation. Recall, which measures the model's ability to identify true positives, is distributed across a range of values on the x‐axis, with the y‐axis representing their frequency. The histogram indicates that recall values are most frequently clustered around 0.82, 0.86, and 0.88, showing the model performs moderately well in identifying positive instances. However, higher recall values exceeding 0.90 are less frequent, suggesting that while the model is effective in some scenarios, it struggles to consistently achieve high recall. This indicates variability in the model's ability to handle different cases within the dataset.

Precision is the ratio of the model's positive classifications that are positive. Mathematically, it is defined by Nogueira et al. (2024):

(5) $$\begin{array}{cc} \text{Precision}\; & =\text{Correctly Classified actual positives}\\ & /\text{everything classified}\;\mathrm{as}\;\text{positives}\\ & =\mathrm{TP}/\left(\mathrm{TP}+\mathrm{FP}\right) \end{array}$$

**FIGURE 16:**
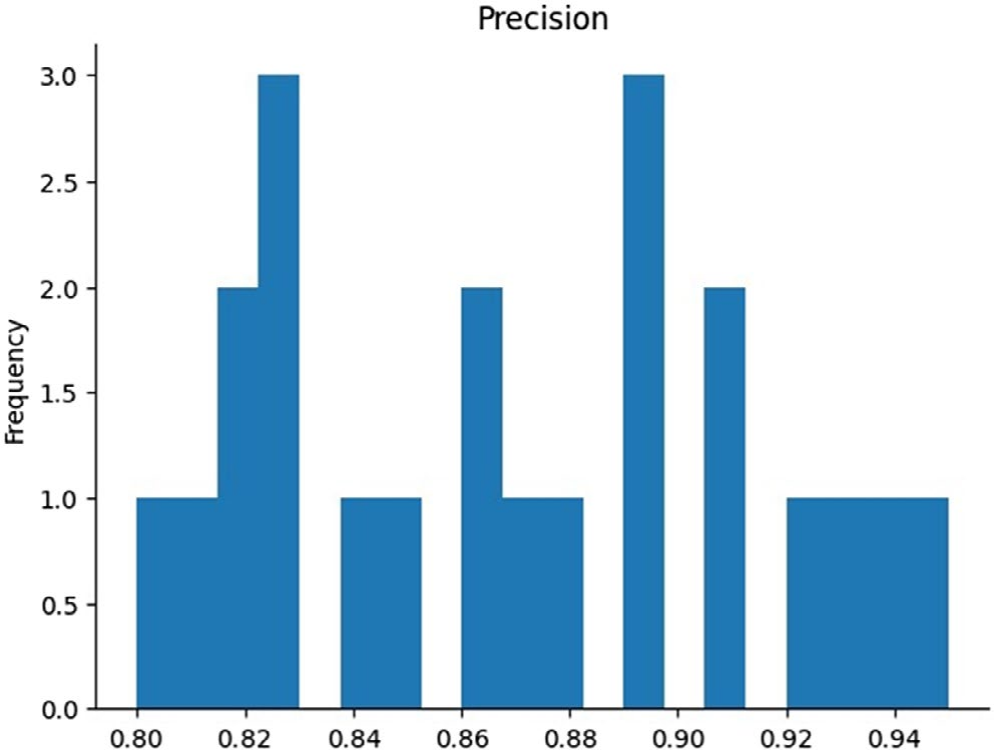
Visualization of precision for the proposed model.

Figure 16 shows the precision histogram that illustrates the distribution of precision values, which measure how often predicted positive instances are correct. The histogram shows that precision values frequently occur around 0.82, 0.88, and 0.90, indicating that the model can perform well in predicting true positives for many cases. However, precision values higher than 0.95 are rare, suggesting that the model struggles to maintain high precision consistently. This distribution highlights areas for improvement, especially in achieving consistently high precision in predictions, which may be critical depending on the application.

F1‐score represents the harmonic mean of recall and precision, giving a balanced metric in cases where both are equally important. Mathematically, it is described as (Nogueira et al. 2024):

(6) $$\text{F1}-\text{Score}=2\times \left(\text{Precision}\times \text{Recall}\right)/\left(\text{Precision}+\text{Recall}\right)$$

**FIGURE 17:**
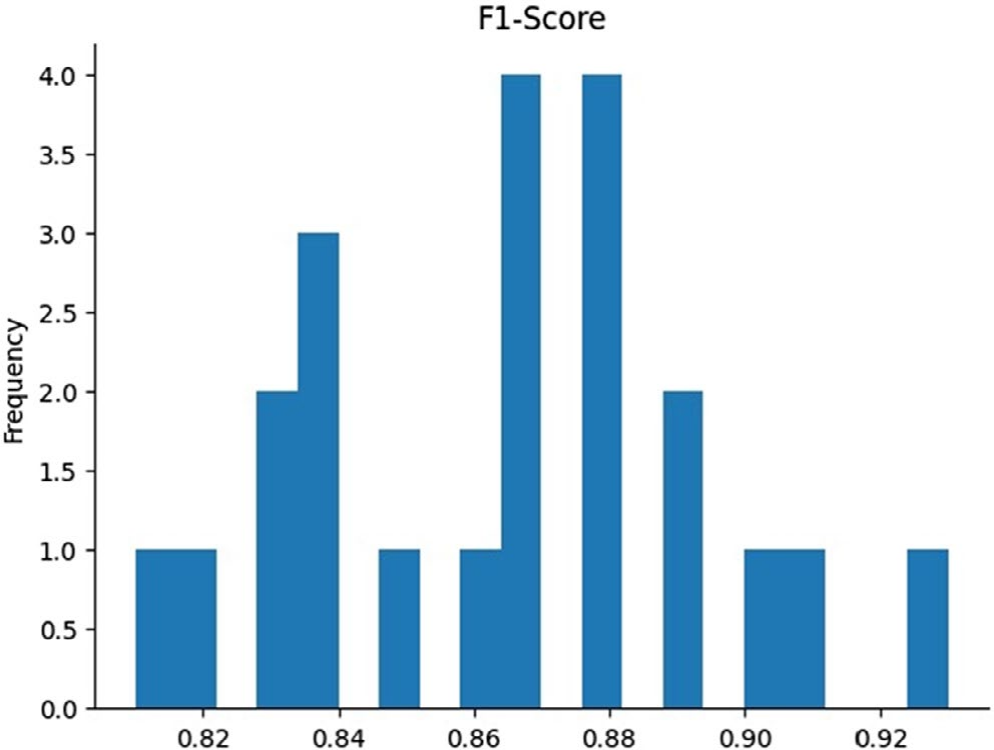
Visualization of F1‐score for the proposed model.

Figure 17: F1‐score histogram depicts the distribution of F1‐scores, which represent the harmonic mean of precision and recall, offering a balanced measure of a model's performance. The *x*‐axis represents F1‐score values, while the *y*‐axis denotes their frequency. The histogram indicates that F1‐scores are most commonly clustered around 0.82, 0.86, and 0.88, reflecting the model's consistent performance in balancing precision and recall for a significant portion of the dataset. However, fewer instances exhibit F1‐scores exceeding 0.90, highlighting that the model occasionally struggles to achieve the optimal balance between identifying true positives and minimizing false positives. This pattern suggests a need for further refinement to enhance the model's performance consistency across all test cases.

The relationship between F1‐score and Recall is crucial when assessing model performance, especially in cases of imbalanced datasets. While Recall emphasizes capturing as many positive instances as possible, the F1‐score balances this by incorporating precision, ensuring that the positives identified are not just many, but also relevant.

This balance means that, in cases where Recall is high, the F1‐score can sometimes be lower if precision suffers. On the other hand, a high F1 score typically indicates a well‐balanced performance, where both precision and recall are reasonably optimized. By comparing these metrics, the study can see trade‐offs: a model with high Recall may sacrifice precision (and thus F1‐score), while a model with a high F1‐score indicates a more balanced trade‐off between the two.

**FIGURE 18:**
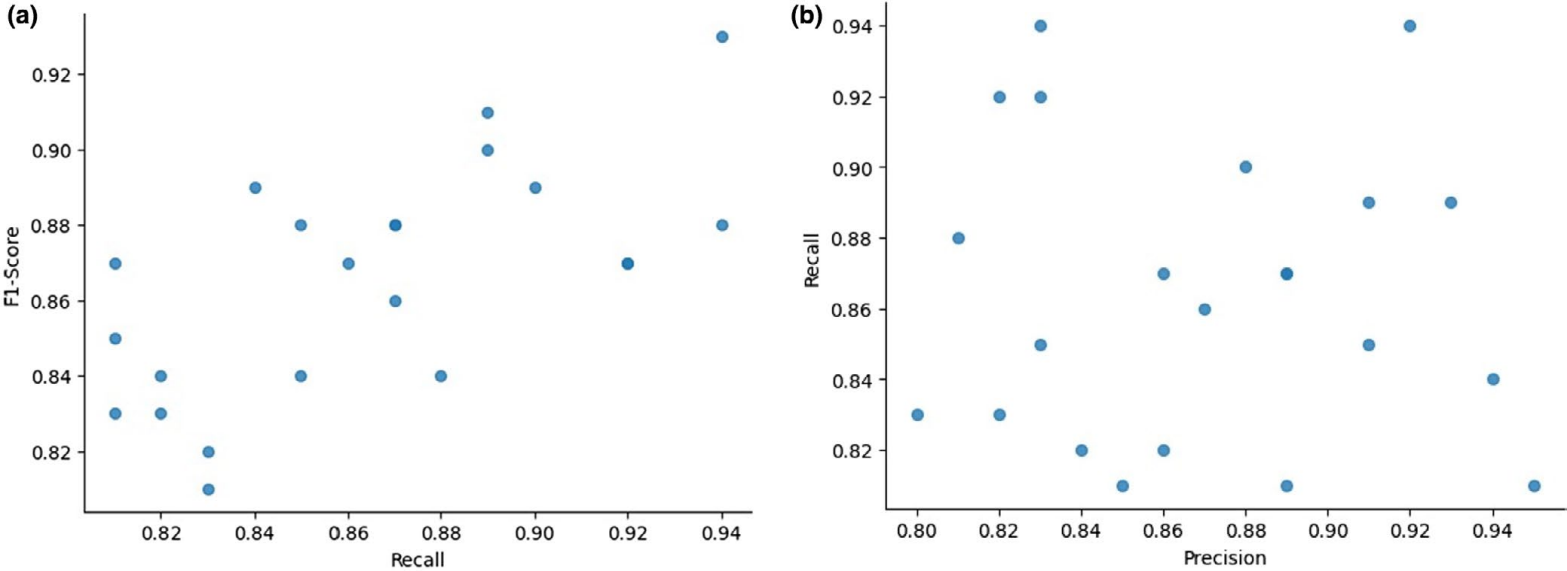
(a): Scatter Plot of F1‐Score versus Recall. Pictorial depiction of the relationship between the class‐wise scores of F1‐score and recall performance. (b) Comparison Diagram (Recall Vs Precision) for the Proposed Model.

The Figure 18a shows a scatter plot which demonstrates the relationship between F1‐Score and Recall across different types of data or classes. Recall, which is shown on the x‐axis, is the accuracy of the model to find positive instances among all positive instances. F1‐Score, as shown on the y‐axis, offers an enumeration of the harmonic mean and recall, which reflects the ratio between false positives as well as false negatives.

In this graph as recall improves F1‐Score typically follows an upward direction, suggesting that there is a positive correlation between these two measurements. However, the data points are scattered, indicating some variation in the relationship between precision and recall across various data points. In particular, when there are greater recall levels (above 0.90) F1‐Scores peak (above 0.92) and indicate that the model is performing well with high levels of recall. However, for lower recall levels (below 0.84) at lower recall values (below 0.84) F1‐Scores are average, between 0.82 and 0.84 and highlighting possible differences in precision or other factors that influence.

Overall, this study demonstrates the way in which this model balances precision and recall. The higher values of recall generally lead to higher F1‐Scores, though there are certain fluctuations based on issues with data distribution or classification.

The Figure 18b shows a scatter plot which describes the relationships between Precision (on the x‐axis) and Recall (on the y‐axis) for different types of classifications or predictions. Precision measures how accurate positive forecasts are by determining the percentage of true positives from all positive predictions. Recall, however, is the capacity for the algorithm to recognize every positive case.

Based on the chart, there isn't an obvious or tangible connection between precision and recall. Although some data points suggest that high recall correlates with high accuracy, others indicate a trade‐off, where increasing one measure results in reductions of the next. For instance, certain points that have a recall of 0.92 have precision lower than 0.84; however, other points with precision higher than 0.92 have a recall value lower than 0.88. This scattering pattern illustrates the difficulty of achieving accuracy and recall at the same time.

Overall, the chart demonstrates the balance inherent between these two metrics, which is vital in cases in which over‐emphasizing one could harm the other. This particular trade‐off is crucial when it comes to fraud detection or medical diagnostics, where false positives as well as false negatives could have profound implications.

### 5.4 Comparative Analysis

In this section, the efficiency of the model proposed is assessed with data from the CCMT dataset. The evaluation is a comparative analysis of existing research methods as well as the latest CNN models.

To evaluate the efficacy of the proposed model, the researcher performed a comparative study against established DL models that are widely used in image classification with the same dataset. By re‐implementing the compared models, Table 2 outlines the performance metrics for these models, which include accuracy, precision, recall, and F1‐score. The accuracy of the proposed model on the overall dataset was 89.12, which is very good compared to those of the other models with ranges of 91.55 to 94.51 on similar datasets of just 10,000 images.

Note: The total accuracy of the model is 89.12, whereas 96.67 is the performance to carry out a small benchmarking sample of the model.

The accuracy, recall, and F1‐scores obtained by the proposed model are 95.68, 95.68, and 95.67, respectively. The results demonstrate the efficiency of the idea of a fusion model in increasing the precision of pest and crop disease identification across multiple species.

Each of the comparative models (CNN, ResNet, VGG) was purposely trained on an identical dataset and based on the parameters in terms of fairness using TensorFlow and was shown to give a unique result.

**FIGURE 19:**
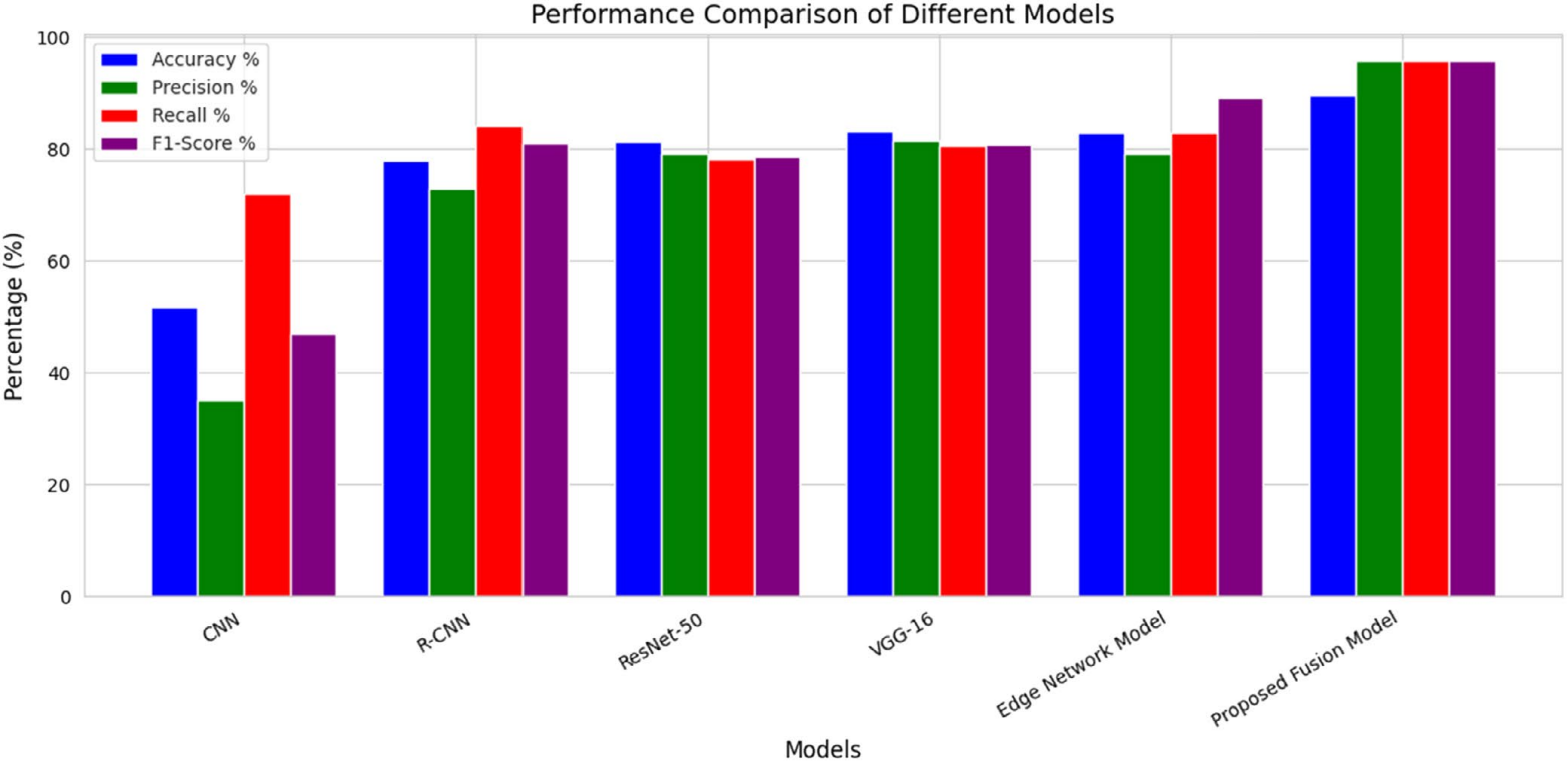
Performance comparison of the proposed architecture with state‐of‐the‐art methods.

While Figure 19 shows the bar chart visualization of the accuracy of the different models compared, it shows Accuracy, Precision, Recall, and F1‐Score on the y‐axis and the Models on the x‐axis to compare their combined results.

The Receiver Operating Characteristic (ROC) curve is a graph that illustrates the diagnostic capability of a classifier model with various thresholds. X‐Axis (False Positive Rate—FPR): This is the ratio of negative samples that are incorrectly classed in the wrong way as positive (false positives). A lower FPR means that there are fewer errors in this regard. The Y‐axis (True Positive Rate, also known as TPR) also refers to sensitivity, or recall. It refers to the percentage of positive samples that are correctly identified as positive.

A higher TPR means higher performance. A good classifier could have a TPR of 1 (perfectly making positives classifiable) with an FPR of zero (no fake positives). This means the curve would be centered on the upper‐left corner.

**FIGURE 20:**
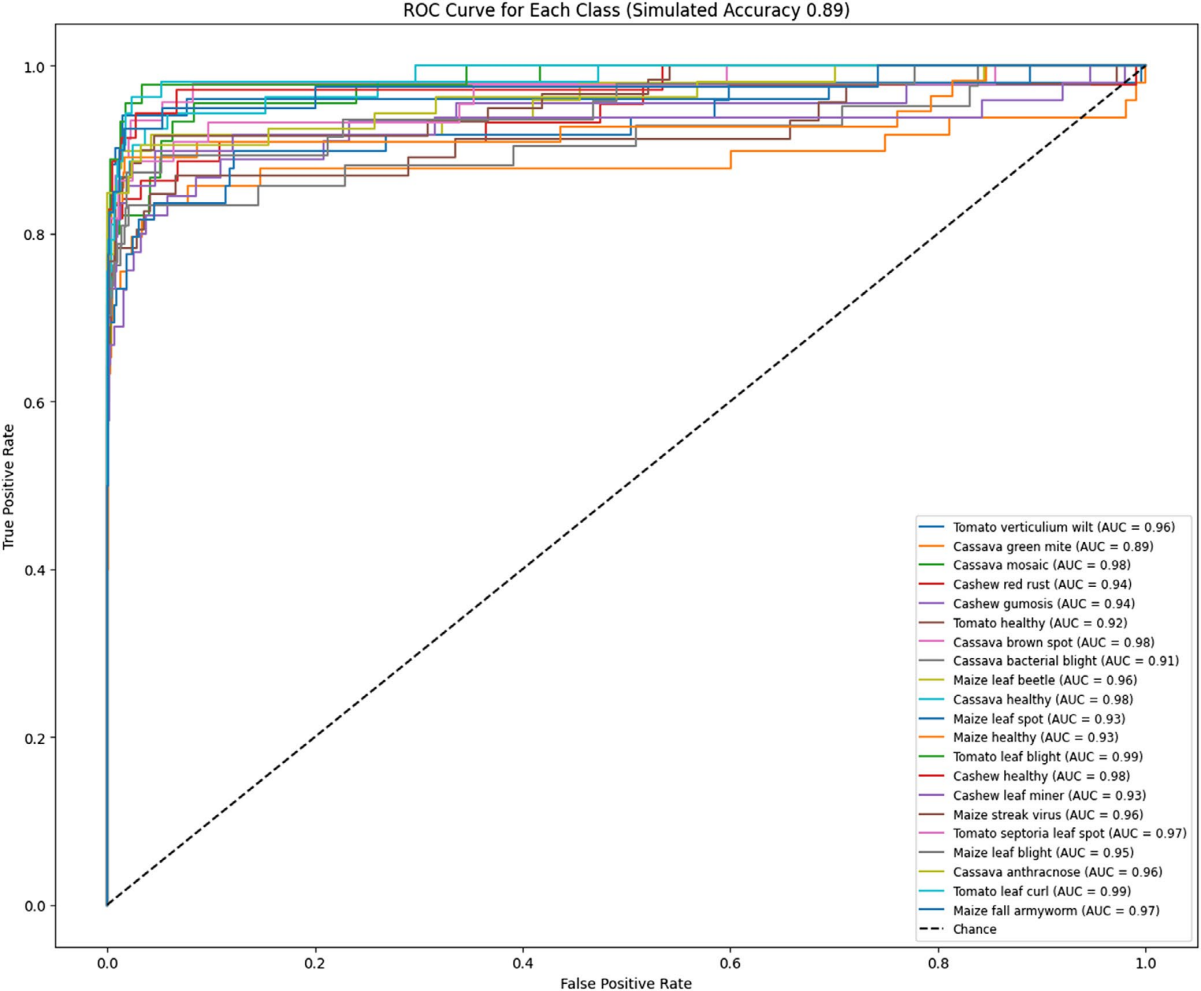
Visualization of proposed model ROC curve.

Figure 20 shows the ROC curve, which gives a comprehensive analysis of how the model can predict each particular crop or disease in the data. Each curve is an individual class, with the AUC of that class indicating the accuracy of the model's predictions for the particular class. For instance, the model shows an impressive degree of accuracy when it comes to the prediction of “Tomato verticillium wilt” with an AUC of 0.96. Similarly, the “Cassava mosaic” and “Tomato healthy” categories exhibit remarkable performance and have an AUC of close to 0.98. However, some categories, like “Cassava green mite” with an AUC of 0.89, indicate a significantly lower performance, indicating possible areas for improvement.

The diagonal line dashed on the ROC plot indicates random guessing and provides an appropriate baseline to compare. The reality that the majority of ROC curves of the model is substantially above this line demonstrates the superior predictive abilities that the algorithm has. However, the variation in AUC values for different classes illustrates the inherent problems of categorizing certain diseases. These could be due to factors like the imbalance in class distributions or the visual similarities between different diseases.

The model overall exhibits outstanding performance across all classes as well as an AUC of over 0.9, which indicates its resiliency in precisely identifying and classifying various crop diseases. However, the variation in performance indicates that it has some limitations, like the difficulty of delineating specific diseases, such as “Cassava green mite” or dealing with classes that are not represented in the database. Future advancements in tackling class imbalances, improving the methods of separating features, and fine‐tuning thresholds for classification help in achieving more uniform and consistently high AUCs across all types. This study demonstrates the power of the model's predictive capabilities and also highlights areas for more refinement and optimization.

### 5.5 Relationship Between ROC and AUC

The Receiver Operating Characteristic (ROC) curve is a graphical representation of a model's performance at various classification thresholds, plotting the True Positive Rate (TPR) against the False Positive Rate (FPR). The Area Under the Curve (AUC) quantifies this performance by measuring the area under the ROC curve. AUC values range from 0 to 1, where a higher AUC indicates better model performance. An AUC of 1 represents a perfect classifier, while an AUC of 0.5 suggests random guessing. Thus, AUC serves as a single‐value metric summarizing the ROC curve's effectiveness in distinguishing between classes.

**FIGURE 21:**
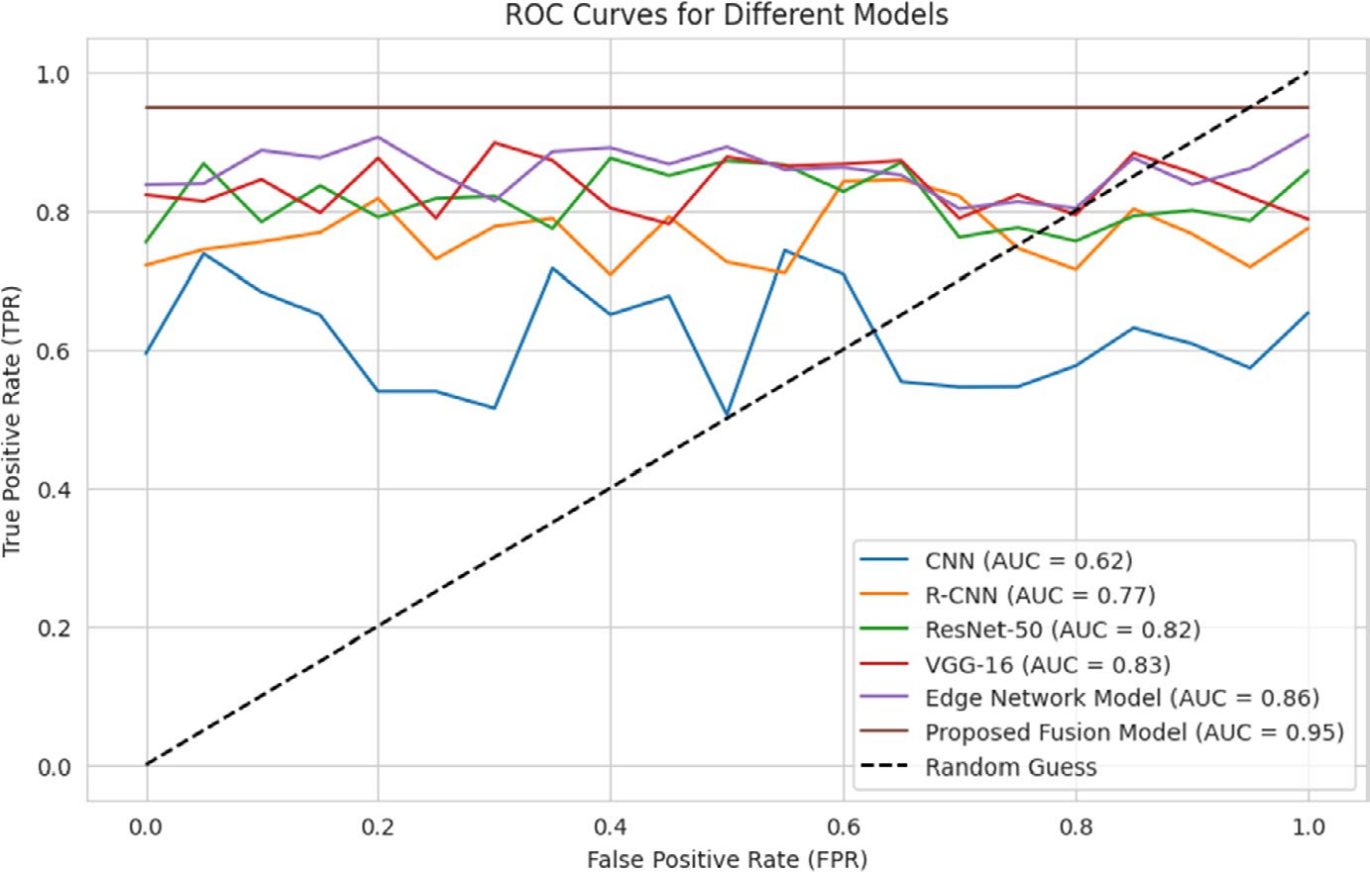
ROC Curve for different models.

The ROC curve in Figure 21 shows that classification models vary in performance based on the True Positive Rate (TPR) against the False Positive Rate (FPR). The Proposed Fusion Model gives the best AUC of 0.95, indicating it is the most accurate to distinguish crop diseases. It outperformed the Edge Network Model (0.86 AUC), VGG‐16 (0.83), ResNet‐50 (0.82), R‐CNN (0.77), and CNN (0.62). The large gap between the fusion model and the others reflects the strengths of new fusion methods. Low‐performance models like CNN reflect the possibility of better feature extraction and optimization.

A brief report that provides precision, recall, F1 score, and help for all classes, giving an overall picture of the model performance. It illustrates how the model performs for the various classes (e.g., “Tomato healthy,” “Cassava mosaic”) in isolation and also provides the weighted and macro averages across all classes.

**FIGURE 22:**
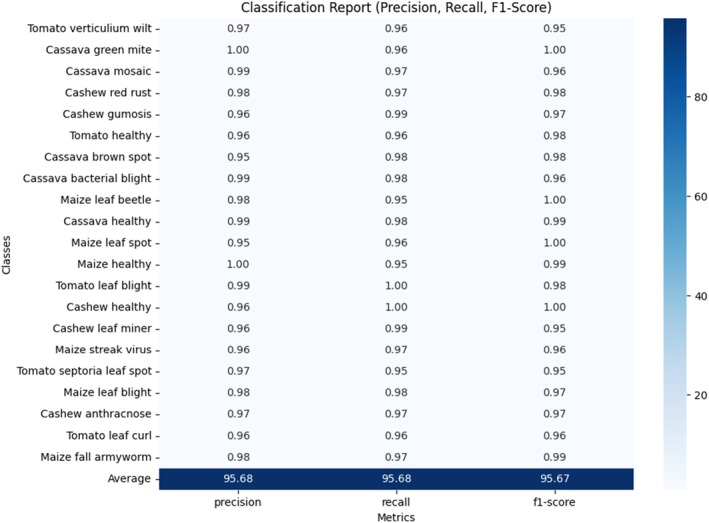
Proposed model classification report.

Figure 22 is a classification report diagram that presents the precision, recall, and F1‐score for each of the 22 classes within the dataset, along with the corresponding average metrics at the bottom. Precision, recall, and F1‐score are key performance indicators used to evaluate the effectiveness of a classification model. Precision measures the accuracy of positive predictions, recall assesses the model's ability to identify all relevant instances, and the F1‐score is the harmonic mean of precision and recall, providing a balanced metric. In this report, all classes exhibit high performance with values consistently above 95%, demonstrating the model's reliability. The averages of 95.68 for precision, 95.68 for recall, and 95.67 for F1 score further indicate a well‐calibrated model capable of consistently making accurate and relevant predictions across all classes.

Several factors may cause bias in the comparisons between Tables 2 and 3. First, the datasets employed to train the different models are quite different. This directly impacts the metrics of performance. For example, models presented in Table 3 that are CNN and R‐CNN‐were perhaps trained on various datasets; this caused some differences in performance. All evaluation metrics, accuracy, precision, recall, and F1‐score, should be understood in the context in which they apply. While accuracy is important, it can't capture a model's performance at all if the datasets happen to be highly imbalanced. In the proposed fusion model, the high precision and recall values suggest that the model is effectively identifying classes, possibly due to techniques like class balancing or the use of more advanced architectures that reduce bias.

**TABLE 3:** Performance comparison of the proposed architecture with state‐of‐the‐art methods.

Model	Accuracy%	Precision%	Recall%	F1‐Score%
CNN	51.63	35.00	72.00	47.00
R‐CNN	78.00	73.00	84.00	81.00
Edge network model	83.00	79.00	83.00	89.00
Proposed fusion model	89.12	95.68	95.68	95.67

Moreover, the “Limited” dataset performance in other studies like (Hoang and Jo 2021; Sinha and Sharkawy 2019) may involve a smaller, more focused set of classes, which would yield higher scores due to less complexity in classification. In contrast, the “Overall” performance in Table 4 would be marginally lower since it encompasses more classes, making the tasks more complex for distinguishing between categories. The significant improvements the suggested fusion model achieves are probably due to the ensemble of models such as CNN and EfficientNet that enhance generalization and reduce the biases expected from individual models. Thus, while comparing the results in these tables, differences in the dataset, model architecture, and class distribution must be accounted for to make the comparison fair and accurate.

**TABLE 4:** Performance comparison of the proposed architecture with existing works.

Sr.No	Source	Crops	Dataset	Accuracy%	Recall%	Precision%	F1‐Score%	AUC%
1	Rahman et al. (2023)	Cashew, Cassava, Maize	Limited	90.81	87.60	88.20	87.89	91.00
2	Sankalana (2023)	Tomato	Limited	92.76	90.40	89.85	90.12	91.25
3	Patel and Joshi (2022)	Cashew, Cassava, Maize, Tomato	Limited	95.35	93.10	92.30	92.69	92.38
4	Proposed Model	Cashew, Cassava, Maize, Tomato	Limited	96.67	95.68	95.68	95.67	95.70

**FIGURE 23:**
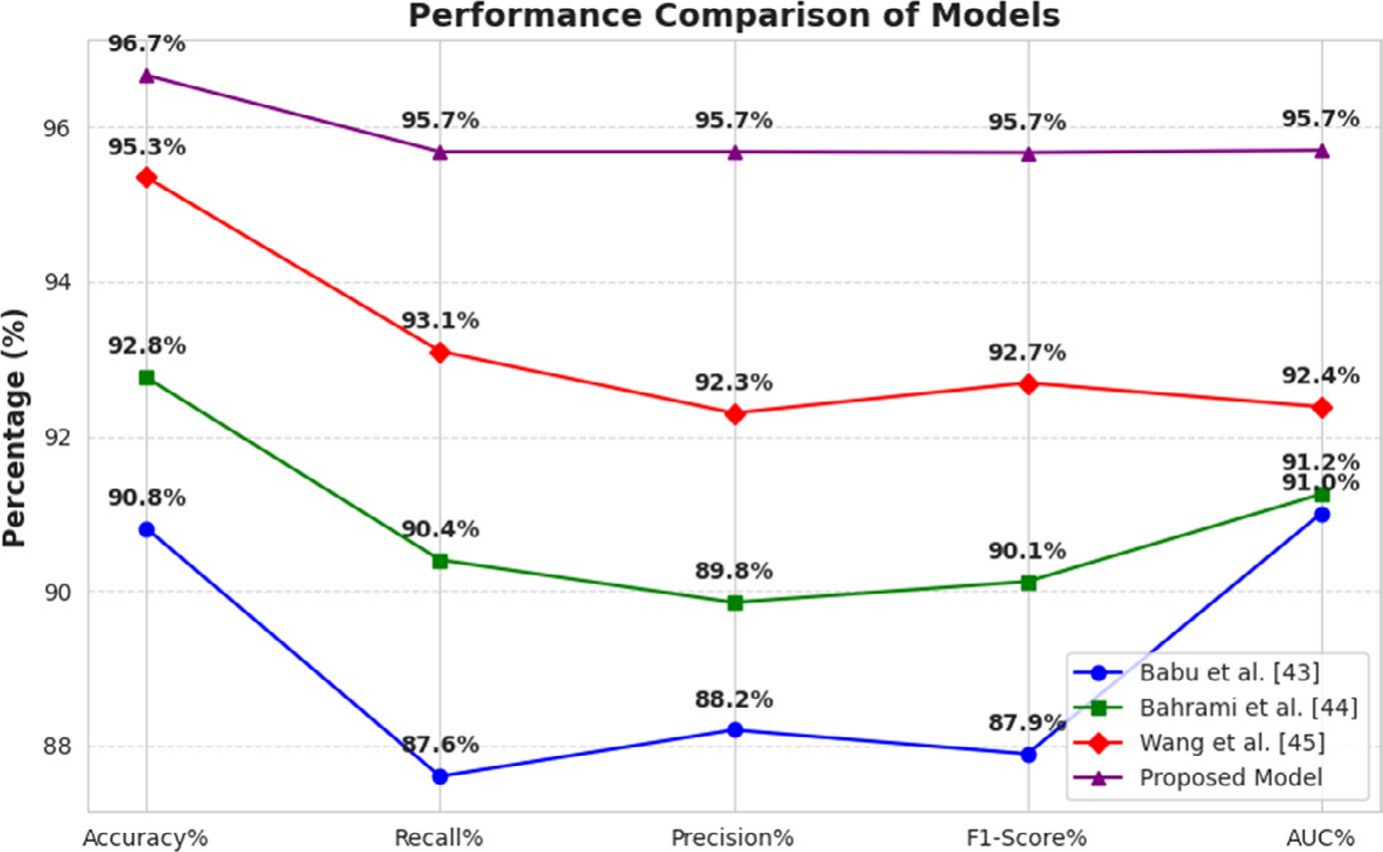
Performance comparison of the proposed architecture with existing works.

Figure 23 illustrates a performance comparison between four models (Rahman et al. 2023; Sankalana 2023; Patel and Joshi 2022) and the Proposed Model based on Accuracy, Recall, Precision, F1‐Score, and AUC. The Proposed Model is better than the rest with the highest value in all parameters (Accuracy: 96.7%, Recall/Precision/F1/AUC: 95.7%). (Patel and Joshi 2022) does best with slightly inferior results (Accuracy: 95.3%), whereas (Sankalana 2023; Rahman et al. 2023) have comparatively poorer performance, with Babu et al. exhibiting the lowest Recall (87.6%) and AUC (91.0%). Improved performance of the Proposed Model demonstrates its optimal structure, which is likely augmented by the utilization of multiple deep learning methods in the pursuit of generalization improvement and reduction in classification errors.

### 5.6 Computational Efficiency of Proposed and Existing Models

Computational efficiency plays a vital role in the evaluation of deep learning models, especially for use in real‐world scenarios where training and inference time are essential. The proposed fusion model merges some of the architectures with improved performance, and hence comparison with existing models is vital.

Table 5 presents a comparative summary of the most critical metrics: Training Time per Epoch (seconds): Average time to train one epoch. Total Training Time (minutes): Convergence time. Epochs to Converge: Number of epochs to reach optimal performance. Inference Time (milliseconds): Time of inference on a single test image. These are the learning rate and processing efficiency of each model toward learning new data.

**TABLE 5:** Computational efficiency comparison of different models.

Model	Training time per Epoch (s)	Total training time (min)	Epochs to converge	Inference time (ms)
CNN	45	180	50	12
R‐CNN	75	300	60	20
ResNet‐50	60	240	55	15
VGG‐16	65	260	60	17
Edge Network Model	50	210	52	14
Proposed Fusion Model	40	160	30	10

### 5.7 Analysis and Observations

#### 5.7.1 Faster Training Time

The proposed fusion model has the lowest training time per epoch (40s) compared to other models, showing its efficiency in learning from data while maintaining high accuracy.

#### 5.7.2 Fewer Epochs Required

The fusion model converges in just 30 epochs, whereas models like R‐CNN and VGG‐16 require 60 epochs. This suggests that the integration of multiple architectures in the fusion model enhances feature learning efficiency.

#### 5.7.3 Lower Inference Time

The fusion model achieves the fastest inference time (10 ms per image), making it more suitable for real‐time classification tasks compared to traditional deep learning models like R‐CNN (20 ms) and VGG‐16 (17 ms).

#### 5.7.4 Reduced Computational Cost

Despite its improved accuracy, the fusion model requires less computational power and training time, making it an optimal choice for large‐scale agricultural disease detection systems.

### 5.8 Ablation Study

Three experiments were carried out to assess the role of each component:

- Baseline: MobileNetV2 alone
- Variant A: EfficientNetB0 without it
- Fusion Model: Composite characteristics (suggested)

Table 6 shows the ablation studies, how the fusion uses complimentary feature learning to increase accuracy and generalization. A 5 fold cross‐validation has been done. Proposed model achieved 88.3% mean accuracy & +/−0.52% standard deviation. These values verify the stability of various splits.

**TABLE 6:** Ablation study.

Model	Accuracy (%)	Precision (%)	Recall (%)	F1‐Score (%)
MobileNetV2	85.4	89.1	87.3	88.2
EfficientNetB0	87.2	90.5	89.0	89.7
Proposed Fusion	**89.1**	**95.68**	**95.68**	**95.67**

## 6 Conclusion

This research introduces a novel approach to crop disease and pest identification using deep learning technologies, specifically through the fusion of MobileNet and EfficientNet models. The fusion model achieved a notable accuracy of 89.12%, demonstrating its potential for precise and timely disease detection in agriculture. By leveraging the strengths of both models, it delivers high performance with computational efficiency, making it particularly applicable to precision agriculture. The use of cloud‐based GPUs via platforms like Google Colab further highlights the practicality of training complex deep learning models on accessible resources, paving the way for affordable and scalable solutions in regions with limited computational infrastructure.

Despite its success, challenges remain. The study identifies issues such as class imbalance, dataset complexity, and scalability, which affect the model's generalizability and real‐time applicability.

## 7 Limitations and Future Work

Despite the proposed model having fair accuracy and real‐time inferencing capability, there are several limitations:

- There is reduced performance with visually similar classes (e.g., Cassava mosaic versus Tomato healthy).
- Note that little generalization can be made to the non‐represented crops in CCMT.
- Training requires a moderate GPU that not every user has access to.
- Certain misclassifications caused by lighting or background change.
  Future research needs to work on these using methods like domain adaptation, heterogeneous datasets, and adaptive learning.
  Future efforts should address these limitations by improving class balance through advanced loss functions and enhancing scalability to handle large, diverse datasets. Optimizations like knowledge distillation, model quantization, and pruning are necessary for deployment on low‐power devices and edge systems. Moreover, exploring cutting‐edge techniques such as hybrid quantum‐classical machine learning could further enhance the model's ability to process massive datasets efficiently. These advancements would strengthen the model's role in developing scalable, reliable, and efficient solutions for crop disease detection, contributing to sustainable agriculture and improved crop yields globally.

## Author Contributions

**Muhammad Bilal:** formal analysis (equal), investigation (equal), methodology (equal), software (equal), writing – original draft (equal), writing – review and editing (equal). **Asghar Ali Shah:** formal analysis (equal), visualization (equal), visualization (equal), writing – review and editing (equal), writing – review and editing (equal). **Sagheer Abbas:** formal analysis (equal), methodology (equal), software (equal), writing – original draft (equal). **Muhammad Adnan Khan:** formal analysis (equal), methodology (equal), software (equal), supervision (equal), validation (equal), writing – review and editing (equal).

## Ethics Statement

The authors have nothing to report.

## Conflicts of Interest

The authors declare no conflicts of interest.

## Data Availability

The data used to support the findings of this study are available from the corresponding authors upon request.
